# Sigma receptors [**σ**Rs]: biology in normal and diseased states

**DOI:** 10.3109/10799893.2015.1015737

**Published:** 2015-06-09

**Authors:** Colin G. Rousseaux, Stephanie F. Greene

**Affiliations:** ^a^ Department of Pathology and Laboratory Medicine, University of Ottawa, Ottawa, ON, Canada; ^b^ MannKind Corporation, Valencia, CA, USA

**Keywords:** Apoptosis, cannabinoids, central nervous system, glutamate, neoplasia, non-opioid receptors

## Abstract

This review compares the biological and physiological function of Sigma receptors [σRs] and their potential therapeutic roles. Sigma receptors are widespread in the central nervous system and across multiple peripheral tissues. σRs consist of sigma receptor one (σ_1_R) and sigma receptor two (σ_2_R) and are expressed in numerous regions of the brain. The sigma receptor was originally proposed as a subtype of opioid receptors and was suggested to contribute to the delusions and psychoses induced by benzomorphans such as SKF-10047 and pentazocine. Later studies confirmed that σRs are non-opioid receptors (not an µ opioid receptor) and play a more diverse role in intracellular signaling, apoptosis and metabolic regulation. σ_1_Rs are intracellular receptors acting as chaperone proteins that modulate Ca^2+^ signaling through the IP_3_ receptor. They dynamically translocate inside cells, hence are transmembrane proteins. The σ_1_R receptor, at the mitochondrial-associated endoplasmic reticulum membrane, is responsible for mitochondrial metabolic regulation and promotes mitochondrial energy depletion and apoptosis. Studies have demonstrated that they play a role as a modulator of ion channels (K^+^ channels; N-methyl-d-aspartate receptors [NMDAR]; inositol 1,3,5 triphosphate receptors) and regulate lipid transport and metabolism, neuritogenesis, cellular differentiation and myelination in the brain. σ_1_R modulation of Ca^2+^ release, modulation of cardiac myocyte contractility and may have links to G-proteins. It has been proposed that σ_1_Rs are intracellular signal transduction amplifiers. This review of the literature examines the mechanism of action of the σRs, their interaction with neurotransmitters, pharmacology, location and adverse effects mediated through them.

## Introduction

Sigma receptors [σRs] are a relatively novel group of receptors originally discovered in the central nervous system [CNS] of mammals in 1976 (1). They represent a ubiquitously expressed unique binding site in the CNS and other peripheral tissues (2–6). σRs are a member of the orphan receptor class for which no endogenous ligand was known until recently – dimethyltryptamine [DMT] (7–9). They also bind with high affinity to several classes of chemically unrelated ligands such as neurosteroids (10), neuroleptics, dextrobenzomorphans [DEX] and several psychostimulants such as cocaine (11), methamphetamine [METH] (12,13) methylenedioxymethamphetamine [MDMA] (14) and methacathinone (15,16). Consequently, it is thought that the σR may mediate the immunosuppressant, antipsychotic and neuroprotective effects of many drugs (17).

Historically, the σR was identified as one of the subtypes of opiate receptors, differentiated using a chronic spinal pain model in the dog, the unique psychomimetic effects induced by N-allylnormetazocine [SKF-10,047] (18) (σ-syndrome), from the effects induced by morphine (µ-syndrome) and ketocyclazocine (κ-syndrome) (1). However, subsequent studies established that σR sites possess negligible affinity for naloxone or naltrexone (19,20); thus, establishing a complete distinction between the non-opiate σ binding sites and the classical µ-, δ- and µ-opiate receptors (21,22). It has recently been suggested that σ_1_R antagonism be used with opioids to increase pain control without increasing the adverse effects of the opioids (23).

Two subtypes of σRs were found originally: sigma-1 [σ_1_R] and sigma-2 [σ_2_R] (24–27). Although another subtype, sigma-3 [σ_3_R], has been suggested, it has not been defined adequately (28,29). σ_1_Rs have been cloned (2), assayed (30) and their biological and physiological roles have been examined more intensively than σ_2_Rs, as until now σ_2_Rs have not been cloned (31).

σ_1_Rs regulate a number of neurotransmitter systems, including the glutamatergic [Glu], dopaminergic [DA], serotonergic [5HT], noradrenergic [NE] and cholinergic [Ch] systems. As these transmitters, which interact with the σ_1_Rs, are involved in many neuropsychiatric disorders their role has been evaluated in a number of these disorders (32). In fact, several lines of evidence have demonstrated that σ_1_R play a role in the pathophysiology of neuropsychiatric disorders such as mood (33), anxiety disorders (34,35) and schizophrenia (9).

Hence, σR ligands are potential therapeutic agents for several neuropsychiatric disorders (36,37). σ_1_R has also been suggested as a target for the treatment of neuropathic pain (38,39) and a treatment for dementia, such as seen associated with Alzheimers disease [AD] (40). In addition, σ_1_R mutations have been implicated in frontotemporal lobar degeneration and motor neuron disease [MND] (41), diseases in which they have been shown to have a low density (42). It appears that there is an association between a variant of the σ_1_R gene and AD (43) where genetic polymorphisms in σ_1_R and apolipoprotein E interact to influence the severity of AD (44).

Many psychostimulant drugs, including cocaine (45) and METH (46,47), interact with σRs in the brain and heart, offering a logical target for medication development efforts (48). σR antagonists and antisense oligonucleotides ameliorate cocaine-induced convulsions, lethality and locomotor activity (49,50), as well as sensitization, and conditioned place-preference in rodents (51). They also reduce alcohol consumption in alcohol-drinking rats (52,53) and Swiss mice (54). Interestingly, the interaction of fluvoxamine [Luvox], a selective serotonin reuptake inhibitor [SSRI], and the σRs may account for its potential amelioration of psychotic depression (55,40), where increased glutamate [Glu] release occurs through activation of serotonin [5-HT_3_] mediated by σ_1_Rs (56), and in patients with schizophrenia (57,40). These findings are supported by research on a depressive phenotype in σ_1_R knockout mice (53). In contrast, the SSRI sertraline worsens the symptoms (58). Not all SSRIs induce their antidepressant activity via the σ_1_R, e.g. paroxetine (59). This detailed review explores the σRs in normal homeostatic and diseased states. First, the structure and function of these receptors are described. Next, sites of σRs, disease states and their relationship to σRs are discussed.

## Molecular biology of σRs

Due to their CNS pharmacological action, most work has been focused on evaluation of σRs in the CNS; however, considerable current research has also been directed toward neoplasia, its treatment and imaging (σ_2_R) (60). σRs are highly expressed in all parts of the brain (25,61,62), where they are predominantly localized in the cell plasma membrane and at the endoplasmic reticulum [ER] of *both* neurons and oligodendrocytes (63). They are dynamically translocated upon ligand binding into cells from the cell membrane (64–66). σ_1_Rs agonists provide protection of the ER from oxidative stress (67).

More recently, a σ_1_R receptor knockout mouse has been developed that displays a depressive-like phenotype, supporting the receptors importance in this psychiatric disorder (53). The database concerning the molecular biology of σRs is large.

### Sigma-1 receptors [**σ**1Rs]

The two subclasses of σR sites (σ_1_R and σ_2_R), distinguished based on their different drug selectivity patterns and molecular weights (21) have no homology to any other mammalian protein (2,68). However, several biochemical features have been observed for σ_1_Rs, such as an allosteric modulation by phenytoin (69) and sensitivity to pertussis toxin or G-protein modulators (70–73), probably though potentiation of opioid transduction independent from receptor binding (74). The σ_1_R site also shows a stereo selectivity with high affinity for the dextro isomers of benzomorphans [BZM], whereas σ_2_R sites show the reverse stereo selectivity with a lower affinity range. 1,3,Di-*O*- tolylguanidin [DTG], 3-(3-Hydroxyphenyl)-N-n-propyliperidin (+) 3-PPP [preclamol] and haloperidol [Haladol®] are non-discriminating ligands with high affinity for both σ_1_R and σ_2_R subtypes (75).

The σ_1_R is a 29 kDa single polypeptide that has been cloned in mice, rats and humans (2,3,6,76,77), the ligand binding profile of which is similar to those described in brain homogenates studies (78,79). The σ_1_R gene, located on chromosome 9, band p13, in human and chromosome 2 in rodents, is approximately 7 kbp long and contains four exons, interrupted by three introns, where exon 3 is the shortest (93 bp) and exon 4 is the longest (1132 bp) (68). Exon 2 encodes 25 kDa membrane proteins for the single transmembrane domain, identified at present, but two other hydrophobic regions exist and one of them may putatively constitute a second transmembrane domain (80).

The σ_1_R sequence contains a 22 amino acid [AA] retention signal for the ER at its N-terminal region and two short C-terminal hydrophobic AA sequences that are probably involved in sterol binding (2). The 223 amino acid sequence of the purified protein is highly preserved, with 87–92% identity and 90–93% homology among tissues and animal species (81). This protein is identical in peripheral tissues and brain, and probably is similar in other tissues as well. It shares a similarity, 33% identity and 66% homology, with a sterol C_8_–C_7_ isomerase (82), but nevertheless is different from any other mammalian protein identified (2,68), outlining the uniqueness of the σ_1_R as compared with any other known receptor.

Hydropathic analysis of the σ_1_R indicates three hydrophobic regions, with some evidence for two transmembrane segments. A crystal structure of the σ_1_R was unavailable at the time of writing, but a 3D model has recently been validated showing agreement of the *in vitro* and the *in* silico model (83).

The σ_1_R gene also has been isolated from human, guinea pig, mouse and rat (2,6,76). AA substitutions in transmembrane domains do not alter the expression levels of the protein but suppresses ligand binding activity (80), suggesting that these AAs belong to the binding site pharmacophore located within the transmembrane domain. In addition, anionic AA residues have been identified that also appear critical for ligand binding (68,77).

Exon-2 codes for a single transmembrane domain present in the σR (68). The fact that the gene for the σ_1_R is located on chromosome 9p13, a region associated with psychiatric disorders (68), helps explain the psychiatric effects of σ_1_R agonists and antagonists.

A splice variant of the σ_1_R has been found in Jurkat cells, an immortalized line of T-lymphocyte cells (84) and in mice (85). Interestingly, σ_1_R-splicing variants have been reported to display σ_2_R characteristics (86,87).

The σ_1_R has been cloned from guinea pig and mouse liver, human placental cell line, and human, mouse and rat brain (2–6). The protein cloned is a 223 AA, 1 transmembrane protein with potent (+)-pentazocine [PTZ], haloperidol, ditolylguanidine (1,3,di-*O*-tolylguanidin) [DTG] and (+)-3-PPP binding, but does not couple with G-proteins (5,76).

At this point, it is not completely clear whether the cloned σ_1_R is the ligand binding subunit of a multi-subunit complex or represents one subtype of the σ_1_R. A study investigating putative transmembrane segments based on homology identified two putative transmembrane segments for the σ_1_R (88). Thus, as research investigates the σRs further, subtypes of the σ_1_R, σ_2_R and possibly the σ_3_R might be found.

Regardless, cloning has led to an important focus on the molecular biology and signal transduction mechanisms of σ_1_R, e.g. inhibition of Ca^2+^ entry into epithelial cells (89). This is discussed in more detail in Sections “σ_1_R ligands” and “Neoplasia”. However, given the one-transmembrane segment cloned, it is most likely that it does not represent the complete functional receptor. More experiments using techniques such as the use of selective σ_1_R gene antisense will elucidate the exact structure of the functional σR in the future (63).

### Sigma-2 receptors [σ_2_Rs]

**Figure 1.**
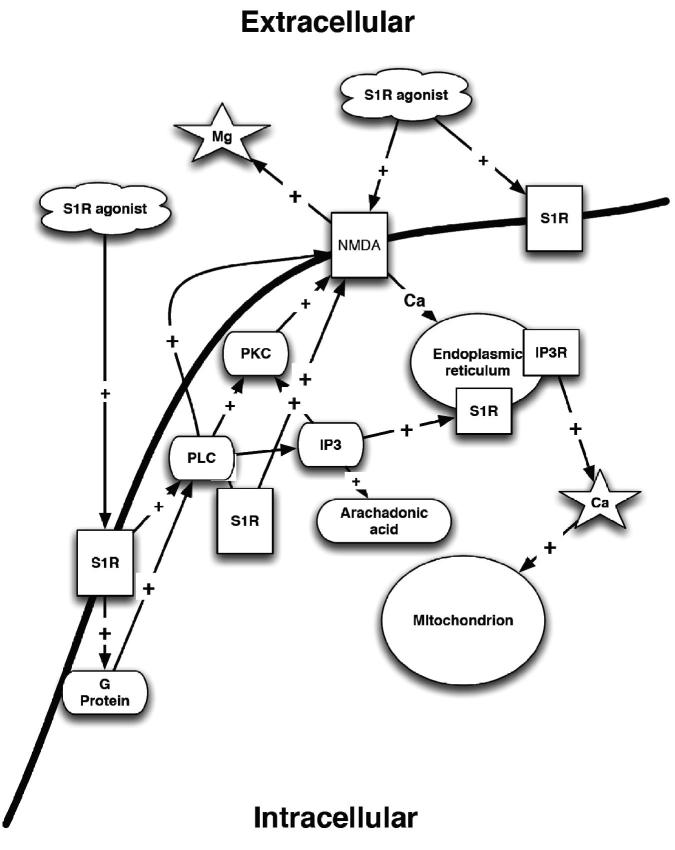
σRs and their effect on intracellular calcium concentrations. PLC – phospholipases C; PKC – protein kinase C; S1R – sigma1; IP3 – inositol triphosphate; IP3R – inositol triphosphate receptor; NMDA – N-methyl-D-aspartate receptor; Mg – magnesium; Ca – calcium.

The σ_2_R site has not been cloned as of yet, but a comprehensive ligand based mapping of the receptor binding pocket has been done (90). The σ_2_R site was first characterized in pheochromocytoma PC12 cells (91), and has a low affinity for (+)-BZM and has an apparent molecular weight of 18 to 21 kDa (92). Some selective and high affinity σ_2_R site ligands are now available such as 1′-(4-(1-(4-fluorophenyl))-1H-indol-3-yl)-1-butyl)spiro (isobenzofuran-1(3H),4′piperidine [Lu 28-179] (93), N-[2-(3,4-dichlorophenyl)ethyl]-N-methyl-2-(1-pyrrolidinyl) ethylamine [BD1008] (92), and ibogaine (94). The site also appears to be important in the modulation of cellular Ca^2+^ concentrations (Figure 1) (95).

Several attributes have been proposed for σ_2_R sites: stem cell differentiation (96); regulation of motor functions (97–99), induction of dystonia after *in situ* administration in the red nucleus (97), regulation of ileal function (100). The sites are also important in the blockade of tonic K^+^ channels (101), potentiation of the neuronal response to *N*-methyl-d-aspartate [NMDA] in the CA_3_ region of the rat dorsal hippocampus (102), or activation of a novel p53- and caspase-independent apoptotic pathway. The mechanism of the induction of apoptosis is distinct from other apoptotic stimuli (103).

The σ_2_R is an σR that preferentially binds to siramesine® (26), selective σ_2_R agonist and also PB28 (104). Activation of the σ_2_R causes apoptosis (104) via triggering of cancer selective cell death signaling (105) by multiple pathways (106). This finding is an important observation for potential antineoplastic drug development. The mechanism by which σ_2_R stimulation induces apoptosis may result from its modulation of intracellular Ca^2+^ stores in some tumors (95). This is of particular importance in those tumors that induce hypercalcemia, e.g. some lymphomas.

The molecular nature of the σ_2_R is still to be fully characterized; however, a structure-affinity and comparative molecular field analysis of σ_2_R receptor ligands has been reported (107). A photo affinity labeling study, using DTG, revealed the existence of two protein bands of MW 25 000 and 21 500 (92). Because the σ_1_R has been cloned (6,77) and shown to be a protein of MW 25 300, it has been presumed that the σ_2_R gene encodes a protein of MW 21 500.

Despite efforts to define the gene for the σ_2_R, it remains unidentified. It has been suggested that the σ_2_R characteristics are, in fact, a consequence of σ_1_ gene alternative splicing (108). However, in the σ_1_R knockout mouse, although σ_1_R-specific drug binding is significantly reduced, binding of nonspecific σR drugs, such as DTG, is not affected, suggesting that the σ_2_R is unaffected (63).

Recently, a novel iodinated σ_2_R ligand (a conformationally-flexible benzamide derivative, 5-bromo-2,3-dimethoxy-N-[2-(6,7-dimethoxy-3,4-dihydro-1H-isoquinolin-2-yl)-butyl]-benzamide, which has 1000-fold selectivity for σ_2_R) has been evaluated as a cell proliferation marker (109).

σ_2_Rs have been implicated in a number of neoplasms, e.g. pancreatic carcinoma (110), urinary bladder tumors (111,112) and breast tumor cell lines (103); therefore, they have been primarily investigated for possible use as cancer chemotherapy targets (113). A more detailed discussion regarding the σ_2_Rs and neoplasia can be found later in the Section “Neoplasia”.

Sigma_3_ receptors [σ_3_Rs] have been proposed (28,29) and were suggested to be linked to the conversion of tyrosine to dopamine [DA] and the activation of protein kinase C [PKC] (114). Here, the proposed σ_3_R agonists may increase the rate of DA synthesis. In addition, putative σ_3_Rs have been imaged in the mammalian brain, and appear to have histamine receptor [H_1_R] properties (115,116). Regardless of these findings, the molecular basis for this diversity is not clear, and the limited amount of literature regarding the subject questions whether the σ_3_Rs really exist, or whether they are a subtype of σ_1_Rs or σ_2_Rs.

## Mechanism of action

σ_1_Rs are intracellular receptors acting as chaperone proteins (46,117). Chaperone proteins assist in the correct folding of other proteins, either during their synthesis or function (118). More specifically, σ_1_Rs modulate Ca^2+^ signaling through the inositol triphosphate [IP_3_] receptor. They dynamically translocate inside cells, hence are transmembrane proteins (118). In fact, it has been suggested that the σ_1_R receptor at the mitochondrial-associated endoplasmic reticulum membrane is responsible for mitochondrial metabolic regulation (119). σ_1_R also promotes mitochondrial energy depletion, Ca^2+^ influx and apoptosis (120). The σ_1_R chaperone protein can be activated or deactivated by specific ligands (121).

These σ_1_R chaperones act at the functional inositol triphosphate receptor [IP_3_R] to the ER and mitochondrion interface to ensure proper Ca^2+^ signaling from ER into mitochondrion. However, under pathological conditions where cells encounter excess stress that results in the ER losing its global Ca^2+^ homeostasis, the σ_1_R translocates and counteracts the potential apoptosis. Thus, the σ_1_R is a receptor chaperone essential for the metabotropic receptor signaling and for the survival against cellular stress (46). σ_2_R is now thought to be a histone binding protein (111).

Although the precise mechanism of the biological response of σRs is still uncertain, it is accepted that σR can modulate a number of neurotransmitter systems, including neurosteroids (49), glutamatergic [Glu] (56), noradrenergic [NA] (122) and dopaminergic [DAergic] ones (26,98) thought to be especially important functional modulators of Glu activity at this site (123–128).

Neurochemical and electrophysiological studies have been crucial in revealing that the σRs regulate the NMDA receptor-mediated glutamatergic, cholinergic and catecholaminergic neuronal responses (26,129,130). σ_1_Rs, at least in part, are intracellular amplifiers creating a super sensitized state for signal transduction (82,131).

### Signal transduction by **σ**Rs

The cloning of a one transmembrane domain σ_1_R, which does not correspond to a G-protein-coupled receptor, reactivated the debate over whether or not σRs act through G-protein-dependent signaling cascades (132). Manipulation of G-proteins alters σR-mediated effects on K^+^ currents (133), acid sensing ion channels (134) and NMDA-evokes release of [^3^H]norepinephrine [NE] (135–137). Yet this manipulation has no effect on K^+^ currents in other models, or on the NMDA response with other σR ligands (138,139). Contrasting evidence exists for the effects of G-proteins on σ_1_R ligand binding (140–143). Therefore, the data concerning the mechanism by which σRs act at the cell membrane level is often conflicting, if not controversial. Given the presumed heterogeneity of the σ_1_R subgroup, it is likely that one subtype of the σR interacts with G-proteins, while another subtype relies on G-protein-independent signal transduction mechanisms, probably via NMDAR.

#### G-proteins

Studies on the modulation of ion channels by σ_1_Rs have made advances in deducing the nature of the signal transduction mechanism (144). It has been suggested, despite the lack of homology between the σ_1_R and classic G-protein-coupled receptors, that σ_1_Rs use G-proteins (74,133,145,146). Accordingly, the σ_1_R could interact functionally with G-proteins through a mechanism that differs from that of classical G-protein-coupled receptors (147). However, many physiological experiments suggest that σ_2_R signal transduction does not involve any G-protein. Experiments on rat neurohypophysis also produced negative results for secondary messenger or G-protein mediation of σ_1_R signaling (138). This finding may be a result of the dose response curve previously described.

In support of σRs’ association with G-proteins, manipulating GTP and 5 guanylylimidodiphosphate [Gpp(NH)p] alters the binding of σR some ligands (70,71,148,149). Contrasting results have also been found for the effects of G-proteins on σ_1_R ligand binding (142,143). Chronic treatments with haloperidol [Haladol®] in rats cause decrease responsiveness to guanine nucleotides following repeated exposure (72). Some selective σR agonists stimulate GTPase activity (132).

The mechanisms of these σR effects are not well understood, even though σ_1_Rs have been linked circumstantially to a wide variety of signal transduction pathways (150). Links between σ_1_Rs and G-proteins have been suggested, but there is also some evidence against this hypothesis (142). Regardless of their involvement of G-proteins, it is more likely that σ_1_Rs act through the NMDAR rather than through these G-proteins (138,139,151).

#### Ion channels and cations

In support of the majority of effects of σ_1_R stimulation being mediated by the ionotropic glutamate receptors [iGluRs], such as the NMDAR, the σ_1_R has been shown to appear in a complex with voltage-gated K^+^ channels, leading to the suggestion that these receptors are auxiliary subunits of the voltage-gated channels (88,138). For example, K^+^ conductance is the prominent target of σ_1_R in rat cortical synaptosomes, C6 glioma cells (101), NCB-20 cells (152), rat neurohypophysis (139) and frog melanotropic cells (133,145).

##### Calcium

An interaction between σRs and Ca^2+^ channels is probable, as (+)-PTZ inhibits the rise in Ca^2+^ levels induced by depolarization of cell membranes and σR ligands decrease basal intracellular Ca^2+^ concentration ([Ca^2+^]*_i_*). This finding supports the hypothesis that the σR activation alone affects [Ca^2+^]*_i_* (2,153,154) and that the σ_1_R is likely coupled to the nicotine-receptor-associated Ca^2+^ ionophore (155).

σR-induced increases in Ca^2+^ currents, which develop progressively following relatively long lasting applications of σR ligands, suggest a direct intracellular coupling of σR to Ca^2+^ channels, through which σR ligands can stimulate voltage-activated Ca^2+^ conductance, independent of the K^+^ channel pathway (156). It is possible that an atypical σ_1_R subtype might also interfere with [Ca^2+^]*_i_* homoeostasis (153,154,157).

In rat sympathetic and parasympathetic neurons, σRs have been shown to modulate high-voltage-activated Ca^2+^ channels including N-, L-, P/Q- and R-type Ca^2+^ channels (158). Although σ_2_R -selective σR ligands were not used, the rank order potency observed, which was haloperidol > ibogaine (an indole alkaloid (159) > (+)-PTZ > DTG, would suggest that this effect may be mediated by σ_2_Rs. In addition to reducing the peak amplitude of the Ca^2+^ current, σRs altered the kinetic properties of these channels.

Several lines of evidence have added further arguments for the involvement of σ_1_R in Ca^2+^ signaling (160). Specifically, the σ_1_R ligands (+)-PTZ and PRE-084 modulate Ca^2+^ signaling in NG108 cells via σ_1_Rs by two different modes of action. Firstly, intracellularly, perhaps on the ER, σ_1_R ligands potentiate bradykinin-induced increase in cytosolic free Ca^2+^ in a biphasic manner, which can be blocked by σ_1_R antisense oligodeoxynucleotide (161), and a second mode of action at the plasma membrane (153,161).

However, the NMDA receptor is probably involved, as such an interaction explains the potentiating action of σ_1_R drugs on NMDA receptor-mediated responses (137,162,163). Further support for this notion is provided by the parallel between their effect on [Ca^2+^]*_i_* mobilization and on the neuronal response to NMDA (135,163,164). It is possible that the major physiological function of the σ_1_R in the CNS is to regulate both types of intracellular Ca^2+^ equilibrium (165).

The changes reported above may cause the reported amplification of Glu, acetylcholine [ACh] and DA responses via the σ_2_R (82,157,164). For example, DTG decreases, whereas reduced haloperidol increases, [Ca^2+^]*_i_* mobilization in colon and mammary adenocarcinoma cells independently of any effect on Ca^2+^ entry through the plasma membrane (153,166). These observations suggest that the biological effect of σ_1_R drugs may be more complex in the regulation of the [Ca^2+^]*_i_* equilibrium; regardless, these results give support to the suggestion that σ_2_R also impacts [Ca^2+^]*_i_* homoeostasis (95,135,153,167).

It has been proposed that the modulation of Ca^2+^ signaling mediated by σ_1_Rs involves the formation of a multiprotein complex, or σ_1_Rs that form multiunit complexes responsible for the modulation of these ion channels (163,165). Specifically, σ_1_Rs have recently been found to anchor ankyrin, a cytoskeletal adaptor protein, to the ER membrane and modulate the function of ankyrin and IP_3_ on the ER (82,164). In this model, the presence of the σR agonist (+)-PTZ leads to the σ_1_R-ankyrin complex dissociating from the IP_3_ (168). This dissociation leads to an increased binding of IP_3_, which in turn increases Ca^2+^ efflux. On the other hand, in the presence of the σ_1_R antagonist NE-100 (156), the σ_1_R dissociates from ankyrin, which remains coupled to IP_3_ on the ER (164).

According to the heterogeneity of the σR subtypes, it has been proposed that in the guinea-pig brain, which expresses mainly the σ_2_R protein, bivalent cations zinc [Zn^2+^], nickel [Ni^2+^], sodium [Na^+^], strontium [Sr^2+^], magnesium [Mg^2+^] and Ca^2+^ inhibit [^3^H]DTG binding in a monophasic manner within a micromolar concentration range (169). However, [^3^H](+)-PTZ binds in a biphasic manner within an mM concentration range, thereby supporting a hypothesis of preferential involvement of the σ_2_R subtype as modulator of Ca^2+^ entry (170). Subsequent dissociation experiments performed with [^3^H]DTG show that verapamil and amidirone, but not nifedipine, BAY-K8644 or amiloride, enhanced the dissociation of [^3^H]DTG from σR-binding sites further supporting the involvement of σ_2_R in the modulation of Ca^2+^ channels.

##### Potassium

K^+^ conductance is the prominent target of σ_1_R in rat cortical synaptosomes, C6 glioma cells (101), NCB-20 cells (152) rat neurohypophysis (139), or frog melanotropic cells (101,133,145,146). An observation has been made that there is interaction between σRs and K^+^ channels. Here the σR ligands DTG and (+)-PTZ inhibit K^+^ currents (133,138,139).

The inhibition of K^+^ channels by σR agonists and antagonists in NCB-20 cells is not affected by pretreatment with A23187, forskolin, phorbol-12,13-dibutyrate, cholera toxin, or pertussis toxin has been shown (152). These results are consistent with the well-known intracellular secondary messenger systems not being essential for the modulation of voltage-gated K^+^ channels by σ_1_R.

Further investigations of this modulation suggest that a protein-protein interaction is the likely mechanism of signal transduction by σRs, as σR ligands do not interact directly with K^+^ channels (88,138), although this effect is enhanced in the presence of σR ligands (138). Therefore, σRs may serve as auxiliary subunits to voltage-gated K^+^ channels in the plasma membrane (88), which also may involve other proteins such as ankyrin and IP_3_R.

Studies on σ_1_R modulation of K^+^ channels, to date, have led to the conclusion that the signal transduction mechanism of σ_1_Rs is membrane independent of G-protein coupling and protein phosphorylation (158) reconstructable in a heterologous system, not requiring cytoplasmic factors, and necessitating the σ_1_R and the K^+^ channel to be in close proximity (138), probably to form a stable macro-molecular complex (88).

Additional studies are required to determine whether the σ_1_R modulation of K^+^ channels is through a direct protein–protein interaction or through intermediate signaling molecules. Given the wide variety of functions that the σ_1_Rs are reported to serve, the most likely explanation is a σ_1_R signaling mechanism involving one or more intermediate signaling molecules, which are localized at or in the plasma membrane, rather than a direct interaction.

#### σ_1_R as an intracellular amplifier

Acute activation of the σ_1_R results in a direct modulation of ([Ca^2+^]*_i_*) mobilization (161,163), and prevents intracellular Ca^2+^ dysregulation in neurons follow an ischemic event. After depletion of intracellular Ca^2+^ from ER stores, the depolarization-induced increase in [Ca^2+^]*_i_* in the cells is modulated by σ_1_R agonists. Both effects are blocked by an antisense oligodeoxynucleotide targeting the σ_1_R (161). Therefore, activation of the σ_1_R results in a complex, bipolar modulation of Ca^2+^ homeostasis.

At the ER level, the σ_1_R activation facilitates the mobilization of IP_3_R-gated intracellular Ca^2+^ pools. This change also occurs at the plasma membrane level. A co-immunoprecipitation study further revealed that the σ_1_R could regulate the coupling of the IP_3_R with the cytoskeleton via an ankyrin B anchor protein, a cytoskeletal protein originally attached to ER membranes (164).

As stated previously, activation of the σ_1_R dissociates ankyrin B from IP_3_R in NG-108 cells, and this dissociation correlate with the efficacy of each ligand in potentiating the Ca^2+^ efflux induced by bradykinin. These results, in conjunction with the σ_1_R subcellular localization (171,165), show that the σ_1_R might act as a sensor or modulator for the neuronal intracellular Ca^2+^ mobilizations and consecutively for extracellular Ca^2+^ influx.

Stimulation of the σ_1_R results in its translocation from the ER (64,163,164), via lipid droplets, to plasma membranes when stimulated by agonists (65,172,173). Thus the translocation of σ_1_Rs at the plasma membrane, associated with the ankyrin B protein consequently affects Ca^2+^ mobilization at the ER (174).

Lipid droplets are formed by coalescence of neutral lipids within the ER membrane bilayer when the coalesced lipids reach a critical size they bud off to form cytosolic lipid droplets, serving as a new transport pathway of lipids between the ER and Golgi apparatus or plasma membrane (65,172,173). Therefore, σ_1_R on the ER may play a role in the compartmentalization of lipids into the ER lipid storage sites and in the export of lipids to peripheries of cells (64).

Lipid rafts play a role in a variety of cellular functions including vesicle transport, receptor clustering and internalization, and coupling of receptors with proteins involved signal transduction (175). Over-expression of functional σ_1_R increases cholesterol contents and alters glycosphingolipid components in lipid rafts of NG108 or PC-12 cells (65,176,177), suggesting that up-regulation of σ_1_Rs potentiates lipid raft formation. Since glycosylated moieties of gangliosides have been proposed to play a role in regulating the localization of growth factor receptors in lipid rafts (175), chronic activation of σ_1_R may present substantial consequences in cell viability and differentiation.

#### Potential endogenous ligand

It has been demonstrated that alterations in endogenous hormonal levels, via adrenalectomy [ADX], castration [CX] (178), ovariectomy [OVX], or pregnancy, affect σR ligands activity when these have been evaluated in the electrophysiological model of the modulation of the NMDA response in the hippocampus (179,180). Similar findings have been seen when investigating the “antidepressant-like” effects of σR ligands in behavioral models of depression (181). Moreover, radioligand binding studies show a 30–40% decrease in [^3^H]SKF-10,047 binding during pregnancy, while ADX/CX enhances [^3^H]SKF-10,047 binding. Subsequent treatment with finasteride, which increases progesterone [PROG] levels, produces decreased [^3^H]SKF-10,047 binding (178,182–184).

Steroid hormones had been original proposed as endogenous ligands of σ_1_Rs, and more recently DMT, a natural tryptamine alkaloid, has been defined as the σ_1_Rs endogenous ligand (7,8). DMT is a hallucinogen found endogenously in human brain. It is commonly recognized to target the 5-hydroxytryptamine 2A receptor [5HT_2A_R] or the trace amine-associated receptor to exert its psychedelic effect. DMT has been recently shown to bind the σ_1_R molecular chaperones, whose function includes inhibiting various voltage-sensitive ion channels (9). Thus, it is possible that the psychedelic action of DMT might be mediated in part through σ_1_Rs.

#### Cell development and plasticity

σR drugs and neurosteroids, acting at the level of the σ_1_R protein, may act in cell development and cell trophic actions (82,185). For example, they have been shown to suppress multiple aspects of microglial activation (186), probably increasing intracellular Ca^2+^. These morphological changes have been previously ascribed to the prominent role of Ca^2+^ in cellular plasticity. This plasticity, which is associated both with the same prerequisite enhancement of NMDA-mediated glutamatergic neurotransmission and protein dephosphorylation that occur downstream from the massive entry of Ca^2+^ into the cell cytoplasm, as well as [Ca^2+^]*_i_* mobilization from the ER and the mitochondria. These events often occur synergistically (187,188).

The amplitude and reliability of both induction and maintenance of long-term potentiation [LTP] in neurons represent an effective model for memory acquisition and consolidation (189). The blockade of LTP and of several learning processes in mice, including spatial learning or passive avoidance, by Ca^2+^ depletion further supports the notion that Ca^2+^ influx and Ca^2+^ compartments are mandatory for memory (187,188). Additional evidence is provided by the poor capacity for acquisition and storage of spatial memory, combined with the lack of hippocampal LTP in transgenic strains of mice lacking subtypes of ryanodine receptors and IP_3_ kinase. This receptor-mediated postsynaptic Ca^2+^ accumulation (Ca^2+^ influx plus massive Ca^2+^ release from internal stores) is reinforced by subsequent activation of kinases such as Ca^2+^/calmodulin-dependent protein kinase II [CaMKII] and PKC (17). Thus, σ_1_R are probably involved in LTP via altering Ca^2+^ influx.

**Figure 2.**
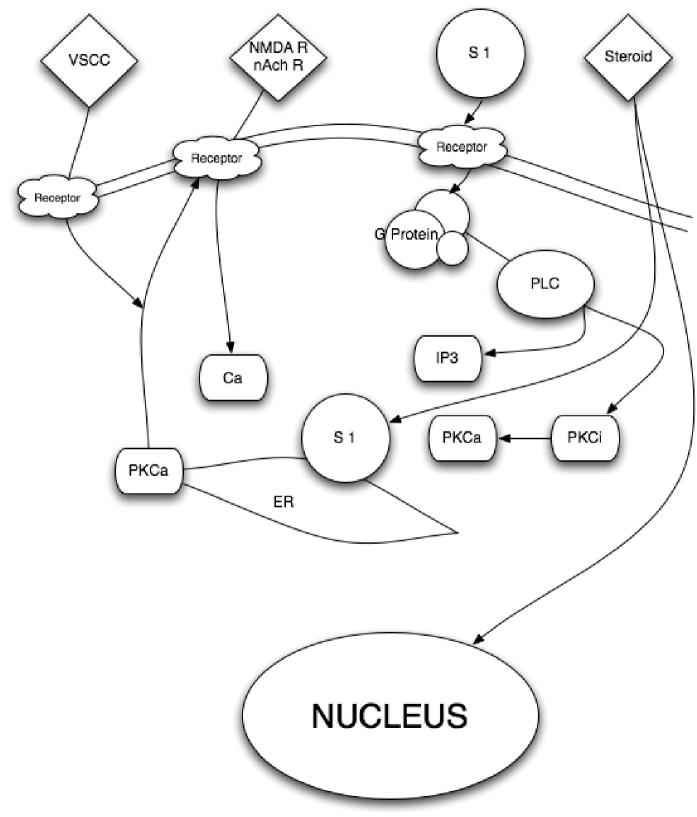
Putative biological action of the σ_1_R on neuronal function. PLC – phospholipases C; PKCa – protein kinase C alpha; PKCi – protein kinase C inhibitor; S 1 – sigma_1_ receptor; IP_3_ – inositol triphosphate; nAch – nicotinic acetylcholine; nAchR – nicotinic acetylcholine receptor; NMDAR – N-methyl-d-aspartate receptors; Ca – calcium; VSCC – voltage-sensitive calcium channels. Once a neuron has been activated, e.g. via Glu or acetylcholine, a concomitant influx of Ca^2+^ and [Ca^2+^]_*i*_ mobilization occur, facilitated by the activation of the endoplasmic-reticulum-bound σ_1_R, which is also triggered by numerous xenobiotics and steroids. The subsequent activation of PLC and the recruitment of the PKCs from its inactive form [PKC_*i*_] to its active form [PKCa], which is translocated to the plasma membrane, result in the activation of various enzymatic processes, as well as the phosphorylation of membrane-bound neurotransmitter receptors. In turn, the σ_1_R translocates to the plasma membrane where it decreases the excitatory neurotransmitter-induced Ca^2+^ influx.

The initial statement that drugs acting via σRs may affect the regulation of [Ca^2+^]*_i_* equilibrium and likely the Ca^2+^ entry through the plasma membrane emerged from *in vitro* binding studies (163,190). The binding studies showed that inorganic Ca^2+^ channel blockers, such as cadmium [Cd^2+^], nickel [Ni^2+^] and Ca^2+^, and the non-selective Na^+^ and Ca^2+^ channel blockers phenylamine, cinnarizine, amidirone and amiloride, reduced the labeling of [^3^H]dextromethorphan (191) and [^3^H]DTG to σR sites (192,193) (Figure 2).

An interesting feature of σRs is that they do not follow the classical pharmacology of a more or less linear dose-response curve followed by a plateau effect. A biphasic bell-shaped dose response curve has been observed for σR ligands in various behavioral, biochemical and electrophysiological paradigms (135,161,182,194). For example, because of the bell-shaped dose response curves, in the electrophysiological paradigm of the modulation of the NMDA response, low doses of σR agonists induce a potentiation of the NMDA response (162,195). At higher doses, the effects of σR agonists such as DTG and JO-1784 progressively decrease and disappear and these molecules act as antagonists by preventing the potentiation induced by low doses of other σR agonists (194).

**Figure 3.**
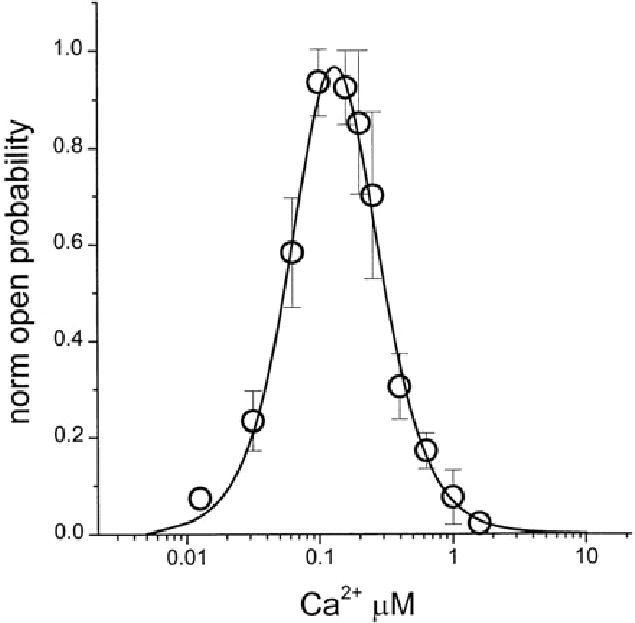
Bell curve dose response. Bell-shaped Ca^2+^ dependence of recombinant IP_3_R. Recombinant IP_3_R activity was measured in bilayers in the presence of 2 µM InsP_3_ and 1 mM Na_2_ATP at cis (cytosolic) Ca^2+^ concentrations in the range between 10 nM and 5 µM Ca^2+^. Ca^2+^ concentration in the cis chamber was adjusted by using calibrated 20 mM CaCl_2_ stock solution and 1 mM mixture of HEDTA and EGTA. Po in each experiment was normalized to maximum Po observed in the same experiment, and then data from three independent experiments were averaged together at each Ca^2+^ concentration (○) (477).

A similar shaped dose response curve has also been described with σR ligands in other models such as in release experiments (135) and in behavioral models (182,183). The exact reason for such dose response curves obtained in so many models have not been well established. It has been proposed that they may be due to the fact that low doses of σR ligands activate one subtype of σRs for which they have high affinity, whereas higher doses may activate another subtype(s) of the σR for which they have a lower affinity. Such activity would counteract the effects observed at lower doses (194,196,197). Nonetheless, it is important to note that the different, and sometimes opposite, results obtained with low and high doses of σR ligands could constitute a very important factor to explain much of the controversy seen in the literature regarding σRs (Figure 3). The importance of the curves seen in these and other experiments will be discussed further on.

Throughout adulthood, differences in the motor changes elicited by drugs affecting σRs are correlated with the number of receptors in the P_2_, and not the P_3_, cellular fraction (198), which supports the hypothesis that translocation of the σ_1_R from the ER to the cell membrane occurs (190). This change decreases with age in motor structures as has been observed in the aged monkey brain where an increase of σ_1_Rs has been found (199).

σR agonists enhance memory performance in young rodents and in rodent models of cognitive impairment (200–205). For this reason, it has been suggested that age-related memory deficits may be responsive to up regulation of the σRs, implying that σ_1_R agonists may have therapeutic potential in dementia (204). In fact, such ability to alleviate memory deficits during aging has also been confirmed in humans for the selective σ_1_R agonist Igmesine® [(+)-*N*-cyclopropylmethyl-*N*-methyl-1,4-diphenyl-1-ethyl-but-3-en-1-ylamine hydrochloride], which appears more efficient among the elderly (206).

Conversely, the σ_2_R subtype exhibits no stereo selectivity and only low affinities for the (+)-BZM (91). It does not appear to be modulated by pertussis toxin-sensitive G*_i_*_/o_ proteins (207), and is predominantly located in the motor system and periphery (21). Clinically, the σ_2_R subtype may be preferentially involved in the motor and anxiolytic effects of σR ligands, as well as in diseases affecting motor and postural control (208). Interestingly, brainstem motor function, which is profoundly sensitive to σR drugs, decreases with age, during which the accuracy and consistency of fine and complex motor performance decrease (208).

The modulatory role of neurosteroids on neuronal function is typified by dihydroepiandrosterone (sulfate) [DHEA(S)] and its effect on σRs (209). NE release induced by NMDA via the stimulation of the σR is significantly enhanced by the addition of DHEAS (210). These findings have been replicated (123,183,210) and the overall data are consistent with the activity of DHEAS as a σ_1_R agonist; hence, neurosteroids potentiate NMDA-induced neuronal excitability (180).

It now appears as though DHEA(S) has an ability to modulate neurotransmitter receptors in the CNS that are primarily involved in learning and memory (209). σR agonists (205) enhance memory performance in young rodents and in rodent models of cognitive impairment (183,200,203,211,212), probably via the NMDAR which is involved in the development of LTP (213–216), an essential element of neural plasticity.

### Activity through neurotransmitters

Neurotransmitters rarely act alone. The delicate balance of the major neurotransmitters, receptors and other methods of transmission control are central to normal homeostasis. These interactions make a reductionist approach to determining the effect of one specific neurotransmitter difficult (217). In fact, experiments that address only one major neurotransmitter may be misleading due to the lack of evaluation of other neurotransmitters and associated receptors.

As σRs are central to a number of CNS and other actions, it is not surprising that they interact with many other concurrent events within and outside the cell membranes on cells of many types. A functional interaction between σR ligands and neurosteroids, such as PROG (218), GluR and opioids, DA and 5-HT exists (98,126–128,183,194,195,219–222).

#### Neuroactive steroids (neurosteroids)

Neurosteroids (223–225), such as PROG, pregnenolone [PREG], dihydroepiandrosterone [DHEA(S)] and their respective sulfate esters PREGS or DHEAS, are involved in regulating the imbalance between excitation and inhibition in the CNS (226); hence, they have been suggested as a treatment for anxiety (227).

**Figure 4.**
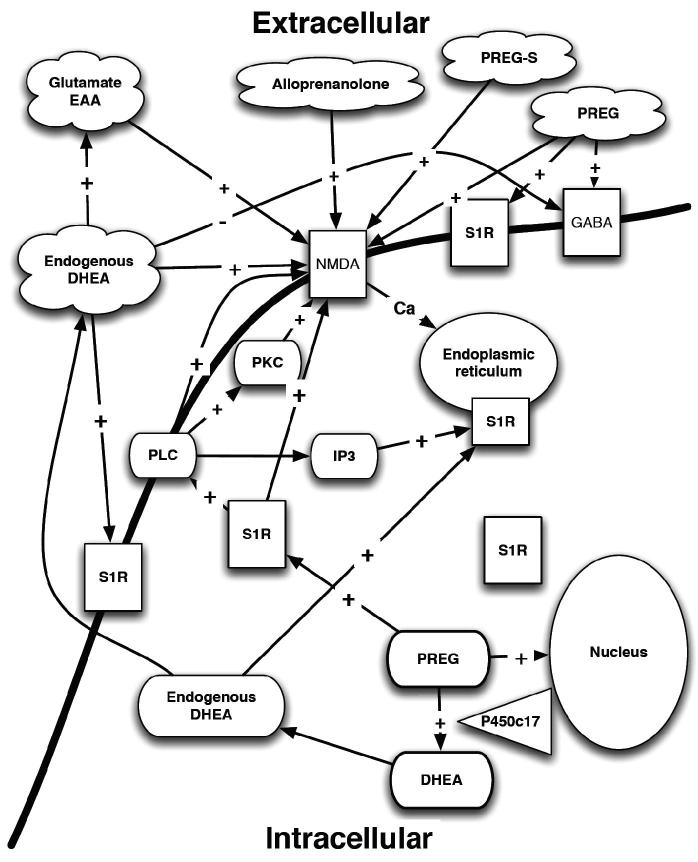
Neurosteroids and their interactions with σRs. PLC – phospholipases C; PKC – protein kinase C; PKCi – protein kinase C inhibitor; S1R – sigma1 receptor; IP3 – inositol triphosphate; EAA – excitotoxic amino acid; GABA – γ-aminobutyric acid; NMDA – N-methyl-d-aspartate receptor; Ca – calcium; DHEA – dihydroepiandrosterone; PREG – pregnenolone; PREG-S – pregnenolone sulfate ester; P450c17 – cytochrome P450 C17.

The initial proposition that steroids behave like endogenous σ_1_R ligands emerged from binding studies (222) and pharmacological experiments (210) leading to the hypothesis that neurosteroids may constitute endogenous ligands for the σ_1_R (2). A functional interaction between σR ligands and neurosteroids, such as PROG (218), GluRs and neurotransmitters exists (Figure 4) (98,194,195,220). Early studies found that neurosteroids bind to σ_1_R (183,228–230), but not to σ_2_R (231). For example, the neurosteroids PROG and DHEA(S) dose-dependently inhibit the *in vivo* binding of [^3^H]-SKF-10,047, an σR agonist, PROG being the most potent (228, 230). These binding data led to the hypothesis that PROG might be the endogenous ligand for σ_1_Rs, which is controversial, as the affinity of PROG for σ_1_R does not appear very high for an endogenous ligand (232). DMT, a natural tryptamine alkaloid, is now recognized as the σ_1_Rs endogenous ligand (8).

The non-neuronal physiological actions of the neurosteroids, demonstrated from embryogenesis through adult life, are mediated secondarily by steroid receptors translocating into the nucleus, and non-genomic neuromodulatory actions affecting directly several ion channels, neurotransmitter receptors and second messenger systems (32). Neurosteroids activate transcription factors; hence, they regulate gene expression and stimulate protein synthesis (233–238). Only the human σ_1_R gene contains a steroid-binding component (239). These neurosteroids are found in the cortex, hippocampus and brainstem, areas of the brain containing high densities of σ_1_R (98).

The neurosteroids 3α-hydroxy-5α-pregnan-20-one (allopregnanolone) [ALLO], allotetrahydrodeoxy-corticosterone, PREGS and DHEAS possess anti-stress, anxiolytic and antiamnesic properties in experimental animal models (212,240–246), and have a possible neuroprotective effect in AD (247). In AD, decreased levels of PREG(S), DHEA(S) and PROG have been identified in the hippocampus (248), cortex and cerebellum, compared to the control animals (249–251). Their actions are mediated via the σ_1_R (Figure 4).

DHEAS and PREGS may also play an important role in depression (252), as decreased levels of DHEA, DHEAS and PREGS have been associated with clinical depression (253), cognitive dysfunction (254,255), dementia (253,256,257) and other neurological conditions (190,258–260). Although there is still controversy as to whether and how the steroidogenic enzymes are involved in the physiology of nervous system (261) and the pathophysiology of neuropsychiatric disorders (262), σRs are critical to their cellular effects.

Clinical investigations in humans have produced evidence for an involvement of neurosteroids in conditions such as fatigue during pregnancy, premenstrual syndrome, postpartum depression, catamenial epilepsy, depressive disorders (252). They possibly alter the expression of conditioned fear stress response in mice (263). However, the exact mechanism underlying the beneficial effects of neurosteroids is not yet fully elucidated (82,263–267).

Modulation of GABA_A_, NMDA, nicotinic, muscarinic, serotonin [5-HT_3_], kainate [Ka], glycine [Gly] and σRs, plus neuroprotection and induction of neurite outgrowth, dendritic spine development, and synaptogenesis are properties of specific neurosteroids (268,269). However, only the human σ_1_R gene contains a steroid-binding component (239), which exerts effects on the genome via individual intracellular steroid receptors (270). Neurosteroids rapidly alter neuronal excitability through interaction with neurotransmitter-gated ion channels, e.g. NMDA.

The 3α-hydroxy ring A-reduced pregnane steroids, ALLO and tetrahydrodeoxycorticosterone, enhance γ-aminobutyric acid [GABA]-mediated chloride [Cl^−^] currents, whereas PREG sulfate and DHEAS display functional antagonistic properties at GABA_A_Rs (271–275). At physiologically relevant concentrations, that is, below 100 nM, these steroids directly activated the GABA_A_R–channel complex (276,277) and exerted a GABAmimetic effect sufficient to suppress excitatory neurotransmission (277).

Certain steroids, including PREG, DHEA, PROG, ALLO and their S (sulfate) esters, rapidly affect neuronal excitability through the modulation of voltage-gated ion channels (278), e.g. voltage-sensitive Ca^2+^ channels [VSCC]s (226,279–283), and neurotransmitter-gated ion channels, such as at the NMDAR level (210,226,284–288). These steroids act at specific extracellular sites that are distinct from one another and from the spermine, redox, Gly Mg^2+^, phencyclidine [PCP] and arachidonic acid sites (289,290). In addition, DHEA(S), but not PREG(S), potentiates the NMDA-evoked catecholaminergic release (210) and firing activity of CA_3_ hippocampal neurons (123). Moreover, the NMDA-stimulates NE release is inhibited by PREGS (210).

It remains unclear whether σ_1_R and neurosteroids exert a common action via the regulation of Ca^2+^ influx and [Ca^2+^]*_i_* regarding amnesic and age-dependent cognitive abilities (163,291). In humans, plasma levels of DHEAS decline with age (258,259), PREG and PREG(S), DHEA and DHEA(S), or PROG decrease in aged mice (292–295) and PREGS decreases in aged Sprague Dawley rats correlating with impaired memory functions (260). However, attenuating effects of DHEAS and PREGS on the conditioned fear stress response are mediated via σ_1_Rs and that PROG has a σ_1_R antagonistic property (263).

It should be noted that σR sites are different from high affinity PCP binding sites, located within the ion channel associated with NMDAR (21). PCP receptor is dependent on the presence of l-Glu; this has led to the suggestion that there may exist an NMDA/PCP receptor complex (296). The lack of selectivity between the σ_1_Rs and PCP binding sites seen following exposure to several compounds, including BZM or PCP derivatives, has led to confusion that was eventually clarified by the availability of new highly selective drugs (212). These compounds are now reference compounds in terms of selectivity between σR and PCPRs.

#### Opioids

**Figure 5.**
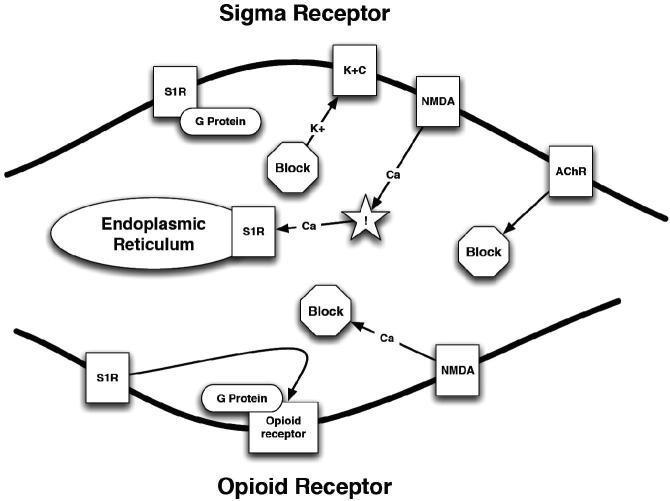
A schematic representation of the opioid receptor and σ_1_Rs. NMDA – N-methyl-d-aspartate receptor; K^+^C – potassium channel; ! – increased concentration; AChR – acetylcholine receptor; S1R – σ_1_R; Ca^2+^ – calcium; K^+^ – potassium.

Opioids have subtle differences in binding to the µ, κ and σRs; however, σRs are a receptor class in their own right (297,298). Although σRs now have been classified as a separate group of receptors from the opioid receptors, the outcome of σR binding is not necessarily different from that when these receptors are bound to opiates (Figure 5) (299), especially since interactions between κRs and σRs have been reported (278,300).

σ_1_Rs have been implicated in the modulation of opioid analgesia. It has been shown that coadministration of a σ_1_R agonist decrease the analgesic power of morphine, whereas the use of an antagonist, DEX, increase analgesia (301), thus illustrating the pharmacological importance of σ_1_R in the brainstem modulation of opioid analgesia (302). Interestingly, the dysphoric and psychomimetic side effects of σRs reside in the levorotatory (*L*) or (−) and not in the dextrorotatory (*D*) or (+)-isomer (303) as exemplified by nalorphine, levallorphan, (−)-PTZ, (−)-3-hydroxy-N-propargylmorphinan and MR 2034. All *L* opiates, produce dysphoria and psychomimetic effects, whereas the *D* isomers of PTZ and MR 2034 do not. Despite this selective response, both (+) and (−) PTZ improve memory via the σRs rather than via the µ and κ opioid receptors *per se* (304–306).

#### Serotonin [5-HT]

**Figure 6.**
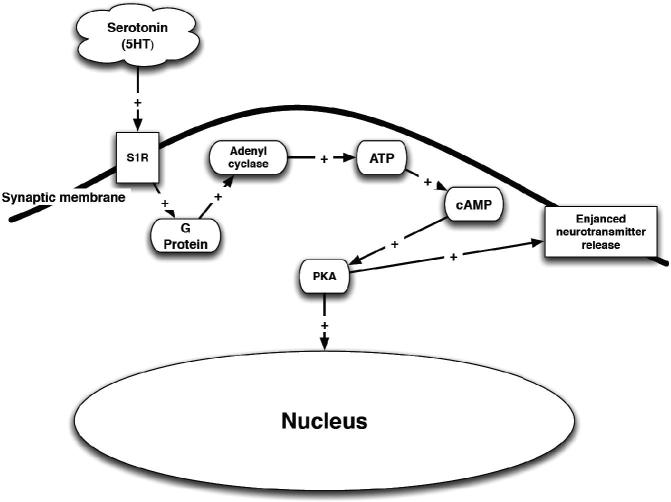
Serotonin (5HT) stimulation of the σ_1_R. PKA – phosphokinase A; ATP – adenosine triphosphate; cAMP – cyclic adenosine monophosphate; S1R – σ_1_R.

There is controversial evidence regarding possible interactions between σR and the 5-HT system (Figure 6). The distribution of 5-HT binding sites in the CNS has been well described (307). These sites include σ_1_Rs. 5-HT and tryptophan (308) play a key role in depression and the mechanism of action of many antidepressants (88,309), probably via a decrease in the firing activity of 5-HT neurons (310–313).

Peripheral 5-HT-σR interactions have been proposed, as DTG, haloperidol and BMY-14802 inhibit the 5-HT evoked contractions of the guinea pig ileum longitudinal muscle and myenteric plexus preparations, showing high correlation with their potency to compete with DTG binding (314). However, the σ_1_R agonist ligand EMD 57445 does not affect 5-HT-related parameters such as 8-OH-DPAT induced behavioral syndrome, m-chlorophenylpiperazine-induced hypothermia or l-5-hydroxytryptophaninduced head twitches (130). In addition, EMD 57445 and the σ_1_R ligand PD144418 do not induce any change in 5-HT or 5-hydroxyindoleacetic acid [5-HI_AA_] levels in various brain regions, suggesting that these ligands exert no effect on 5-HTR populations or 5-HT metabolism (130,264). Interestingly, EMD 57445 and PhmD 144415 have been suggested to be σ_1_R antagonists.

Whether or not σR ligands can modulate 5-HT neuronal activity *in vivo,* the effects of short- and long-term administration of various σR ligands on 5-HT basal neuronal activity in the dorsal raphe nucleus [DRN], have shown that acute treatments with SSRIs and MAOIs induce a decrease in the firing activity of DRN 5-HT neurons (310,312,313,315). There is an eventual restoration of the firing activity of these neurons (312,313,316,317) due to the desensitization of the 5-HT_1A_ autoreceptors in the CNS (311,318–321).

In contrast to what has been observed in the dorsal hippocampus, acute iv administration of (+)-PTZ has no effect in the DRN. Interestingly, however, the σR ligands, 4-IBP, (+)-PTZ and DTG, after either two or 21 days of treatment induce a 50% increase in the firing activity of 5-HT neurons of the DRN (322). These findings suggest modulation of 5-HT neurotransmission by σR ligands *in vivo*, a novel finding with respect to σR research, again supporting a role for σR in depression, probably mediated by σ_1_Rs via the 5-HT_1A_ receptor (323–325).

Interestingly, OPC-14523, a σ_1_R agonist, decreases the responsiveness of the 5-HT_1A_R after two days of treatment (326,327). This is particularly significant given that classical antidepressant medications require chronic treatment for this decreased receptor response to occur (313,319,320). If this effect is shown to be a general effect, present with other σ_1_R agonists, the rapid desensitization of the 5-HT_1A_ autoreceptor, in addition to the observed rapid increase in the firing activity of 5-HT neurons after only two days of treatment with σ_1_R agonists, would constitute another argument to suggest that σR agonists have potential to produce a fast onset of antidepressant effect.

The neurosteroid σ_1_R agonist PROG does not have any effect by itself on 5-HT neuronal activity in the DRN, but several of its metabolites, such as ALLO or DHEA, increase the firing activity of DRN 5-HT neurons. Interestingly, at least part of the effects of neurosteroids is mediated through an activation of σRs as they are reversed by NE-100 (328).

The precise mechanism by which σR ligands increase the firing activity of DRN 5-HT neurons has not been established. One possibility is that the effect is mediated locally, in the DRN, as a consequence of the modulation of the Glu neurotransmission, since AMPA and NMDA GluRs have been shown to mediate glutamatergic excitatory input in the DRN (329).

The σ_1_R-mediated effect on firing could also be an indirect one, as σR ligands rapidly modulate NMDAR-mediated transmission in the hippocampus, which leads to a modulation of 5-HT neurotransmission in the DRN via feedback loops to DRN 5-HT neurons. In fact, an afferent connection has been identified that projects from the hippocampus to the DRN via the lateral habenula (330–336), and the long feedback loop that projects from the DRN to the prefrontal cortex [PFC] and back to the DRN (329,333,335,337–340).

Therefore, the activity of σRs on the DRN neurons is dependent on the balance between the excitatory input (the Glu system) from various brain regions (e.g. lateral habenula and mPFC) and inhibitory input from GABAergic interneurons in distal areas (e.g. periaqueductal gray area) and local GABAergic interneurons situated in the DRN (341,336).

Another factor likely contributing to the requirement of a sustained treatment of σR agonists to observe an antidepressant effect is based on the density of σRs at the plasma membrane, which is progressively altered by the presence of σR ligands. σR agonists induce an increase in the σR density at the plasma membrane following a minimum of two days of treatment (185) exerting effects on NMDAR-mediated signaling.

#### Dopamine

The σ_1_R subtype is involved in the facilitation of cortical Dopamine [DA] transmission in the rat brain (342). σ_1_Rs are located in limbic areas, including nucleus accumbens [NAC] (343) and PFC, both of which are thought to be involved in schizophrenia (344). Many antipsychotics, including haloperidol (345), bind with high affinity to σ_1_Rs, where the DAergic hyperactivity in the NAC is thought to underlie positive symptoms of schizophrenia (including delusions, disordered thoughts and speech, and tactile, auditory, visual, olfactory and gustatory hallucinations, typically regarded as manifestations of psychosis), while DAergic hypoactivity in PFC the negative symptoms (including deficits of normal emotional responses or of other thought processes). σ_1_R ligand agonists increase extracellular DA levels in rats (346) whereas their antagonism inhibits DA-induced abnormal involuntary movements (347).

σRs regulate NMDA-[^3^H]DA release in caudate-putamen [CP], the neuroanatomical substrate for extrapyramidal side effects resulting from chronic 2-amino-7-phosphonoheptanoic acid [AP-7] treatment (348). In that study, in the NAC, regulation of DA release by the prototypical σR agonist (+)PTZ mediated predominantly by the σ_1_R, whereas in the PFC a portion of the (+)PTZ effect is likely mediated by the σ_2_R.

In both the NAC and PFC, regulation of DA release by the σR agonist BD737 is mediated primarily by the σ_1_R, not via the opioid receptors, the NMDAR-operated cation channel, or by σR effects upon [^3^H]DA accumulated by noradrenergic terminals in PFC (349). In fact, the action of NMDA in primary cortical neurons is regulated differently by ligands with differential affinities at DA D_2_ and σRs (291).

The effects of different selective σR ligands on DA and Glu-NMDA neurotransmissions, both in origin (A10 and A9 areas) and terminal NAC and CP regions of the rat mesolimbic and nigrostriatal DA-ergic systems, have been evaluated. The selective σ_1_R ligands 2-[4-(4-methoxy-benzyl)piperazin-1-yl-methyl]4-oxo[4H]-benzo-thiazolin-2-one [S-21377] and 2[(4-benzyl piperazin-1-yl) methyl] naphthalene, dichlorydrate [S-21378] slightly increase the spontaneous firing rate and potentiate the NMDA-induced neuronal activation of DA-ergic neurons in the A9 and A10 regions. (+)N-cyclopropylmethyl-N-methyl-1,4-diphenyl-1-ethyl-butyl-2-N [JO-1784], another selective σ_1_R ligand, has produced no or little effect in these areas (350).

A selective σ_2_R ligand 1,4-bis-spiro[isobenzofuran-1(3H), 4′-piperidin-1′yl]butane [Lu 29–252] significantly potentiates the NMDA-induced increase in firing activity of A_10_ DA neurons. Functional interaction between σ_2_R and NMDARs in the A_10_ region has been reported (350); thus, DA release in the striatum may be modulated by multiple σR subtypes. In such a situation, NMDARs may mediate the stimulatory effect of σR ligands on DA release in the striatum (351).

In addition, σR may regulate the release of DA along with an action at the NMDAR, e.g. the pharmacological effects of amantadine on DAergic transmission are proposed to result from an uncompetitive antagonism at this receptor (352). These data demonstrate that aminoadamantanes behave as σ_1_R agonists, and confirm an involvement of this receptor in modulating DA receptors exerted by therapeutically relevant concentrations of amantadine (352,353).

The regulation of DA release is much more complicated than has been alluded above. Regardless, work has showed that activation of σ_2_R results in the regulation of dopamine transporter [DAT] activity via a Ca^2+^- and PKC-dependent signaling mechanism (354).

#### Nicotine and acetyl choline [ACh]

σ_1_R ligands noncompetitively inhibit nicotine-stimulated catecholamine release from bovine adrenal chromaffin cells in a concentration-dependent and reversible manner (355). The rank order of potency of ligands to inhibit nicotine stimulated catecholamine release is correlated with that observed in radioligand binding assays selective for the σ_1_R subtype. This naltrexone-insensitive effect is paralleled by an inhibition of nicotine-stimulated increases in [Ca^2+^]*_i_*. σR ligands are without effect on catecholamine release or [Ca^2+^]*_i_* in the absence of nicotine (155), although the inhibitory effect of σR ligands on the nicotine-evoked Ca^2+^ uptake is not directly coupled with either the σ_1_R or σ_2_R sites (356).

Nicotine accelerates the association of the receptor selective radioligand, [^3^H](+)PTZ, to adrenal medullary homogenates while having no effect on the rate of ligand dissociation, consistent with a σR ligand binding site closely associated with and allosterically modulated by the nicotinic acetylcholine receptor [AChR] (155). Thus, the actions of agonists at the nicotinic AChR are modulated by σ_1_R selective ligands (160). In addition, the increased ACh level seen in rat frontal cortex induced by (+)N-allylnormetazocine supports the activity of σRs in ACh regulation (357–359).

#### Nitric oxide

It has been shown *in vitro* that σR ligands prevent Glu-induced activation of nitric oxide synthetase [NOS] (360). Nitric oxide [NO] is an important mediator in ischemic brain injury (361–363), and in many other disease states. Specifically, NO derived from constitutively expressed NOS in neurons [nNOS] and the inducible isoform expressed by many cells [iNOS] are important in excitotoxic injury cascades (363,364), such as can be seen following exposure to EAAs. Pharmacologically selective inhibitors of nNOS and iNOS, such as the σ_1_R (365), attenuate infarction volume after focal cerebral ischemia (362,366,367).

A potent σ_1_R infusion into normal striatum by microdialysis attenuates basal, and NMDA-evoked, striatal NO production *in situ* (368); therefore, it is not surprising that systemic σR ligand treatment reduces stroke damage by preventing ischemia-induced NO production (369) with reduced infarct volume (370). These findings have been reproduced more recently (187). For this reason it has been suggested that σ_1_R agonists should be considered as neuroprotective drugs, where some of the protection offered occurs through inhibition of inducible NOS (365).

#### Glutamate [Glu]

Although many AAs play a role in neurotransmission, Glu, Gly and GABA are among the more common and better-understood neurotransmitters (371–375). Glu mediates an estimated 50% of all the synaptic transmissions in the CNS. Glu, glycine and GABA are metabolic intermediates and neurotransmitters, where Glu is the major excitatory neurotransmitter, and Gly and GABA are the major inhibitory neurotransmitters (326,350,376–380). Glu is involved in nearly all aspects of normal brain function including learning, memory, movement, cognition and development (381–390).

Glu is synthesized, stored and released from the presynaptic terminal, has specific neurotransmitter receptors are localized on the postsynaptic cells, and is eliminated from the synaptic cleft by specific transporters. In addition to Glu, aspartate [Asp] also acts as a major excitatory neurotransmitter (382,391–395) by stimulating or exciting postsynaptic neurons.

**Figure 7.**
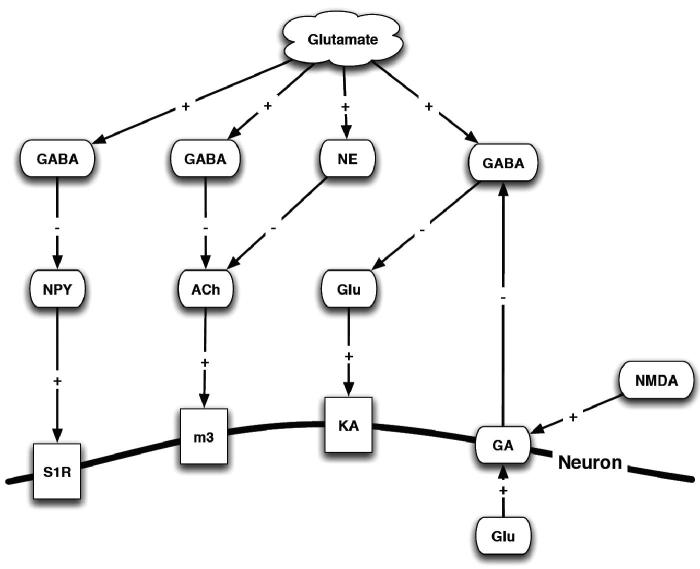
Interaction of glutamate, neurotransmitters and the σR. NMDA – N-methyl-d-aspartate receptor; NE – norepinephrine; NPY – neuropeptide Y; ACh – acetylcholine; M3 – rat muscarinic acetyl choline receptor; GABA – γ-aminobutyric acid; GA – Ga-binding protein α-chain; Ka – kainate; Glu – glutamate; S1R – σ_1_R.

From Glu labeling studies, the average concentration of Glu in ganglion cells is 5 mM (396). Physiological studies using isolated cells indicate that only µM levels of Glu are required to activate GluRs (397–399). Thus, the amount of Glu released into the synaptic cleft is several orders of magnitude higher than the concentration required to activate most postsynaptic receptors. As σRs seem to mediate a number of processes through the Glu system, a more detailed discussion of the Glu system is provided (Figure 7).

N-Acetyl-aspartyl-glutamate [NAAG] is abundant in the mammalian CNS, which has led to the hypothesis that this dipeptide is the storage form of Glu (400,401). Brain tissue has a remarkable ability to accumulate Glu, an ability resulting from Glu transporter [GluT] proteins present in the plasma membranes of both glial cells and neurons (402). Glu is at the center of other metabolic events, e.g. Glu serves as substrate for the synthesis of N-acetyl Glu, an essential allosteric activator of carbamyl phosphate synthetase I, a key regulatory enzyme in the urea cycle (403). It has a well-described transdeamination system involving aminotransferases and Glu dehydrogenase, where Glu plays a key catalytic role in the removal of α-amino nitrogen from AAs. Finally, the “Glu family” of AAs (arginine, ornithine, proline, histidine and glutamine) requires the conversion of these AAs to Glu for their metabolic disposal. The Glu system is probably the mediator of excitatory effects seen following σRs stimulation (404) by σR agonists such as phencyclidine [PCP] (201).

At toxic concentrations, Glu acts as a neurotoxin (excitatory amino acid [EAA]) capable of inducing severe neuronal damage and necrosis by causing over excitation of neurons through receptor-mediated depolarization and Ca^2+^ influx (373,405–411). However, Glu is not the only EAA that can cause excitotoxicity and cell death in the CNS (382,391,392,394,412,413). The σ_1_R ligand PRE-084 protects against excitotoxic perinatal brain injury in newborn mice (414), indicating a central role for the σRs in modulating the excitatory effects of Glu.

Other EAAs access the brain tissue of the circumventricular organs located outside the blood brain barrier [BBB] (415–420). An array of GluRs are known to be present on pre- and postsynaptic membranes that are used to transduce integrated signals using an increased ion flux and second messenger pathways (382,389–392,421,422,). It is the excessive activation of these receptors that leads to neurotoxicity, often referred to as “excitotoxicity”.

There are five main factors necessary for the transition of Glu and Asp from neurotransmitters to excitotoxins, including inadequate neuronal ATP levels, inadequate neuronal levels of Mg^2+^; high concentrations of inflammatory prostaglandins; excessive free radical formation (423,424) and inadequate removal of synaptic Glu (296,373,408,425,426). It has been postulated that excitotoxicity is involved in the pathogenesis of many types of acute and chronic insults to the CNS (416) and peripheral tissues (418), and interestingly, excitotoxicity has also been suggested as a central mechanism in fluoride neurotoxicity (427).

Glu and its structural analogues may enter the food supply during preparation or processing as contaminants or additives in its free form or bound to peptides and proteins (428–437). These analogues include monosodium Glu [MSG], l-aspartate, l-cysteine, related sulfur AAs, B-*N* -oxalyamino-l-alanine [BOAA or ODAP], B-*N*-methyl-amino-l-alanine [BMAA] and the seafood toxin domoic acid [DomA] (429,432,435,438–440). Structurally similar environmental dietary excitotoxins (441), such as DomA, one of the most potent neurotoxins in seafood can enter our food supply (439). Contamination of mussels by sea diatoms producing DomA (429–431,442), results in neuronal excitation resulting in severe seizures (429–431,433,434,439,442). Survivors of severe cases suffered permanent loss of short-term memory, a phenomenon that lead to the term amnesic shellfish poisoning (415,418,431,434,437,439).

It now is clear that the σRs are important in modulating Glu-mediated seizures (443), and protects neurons against Glu toxicity *in vitro* (444), although direct interaction with NMDARs should not be forgotten as a crucial element in the neuroprotective effects of σRs ligands with affinity for NMDARs (445,446).

Although excitotoxic effects can be pronounced during acute events such as ischemic stroke and trauma, they can occur in prolonged chronic neurodegenerative diseases such as AD (425), Parkinsons disease [PD] (447), Huntingtons disease [HD] (448) and Amyotrophic Lateral Sclerosis [ALS] (373,449) schizophrenia, anxiety, depression (425,450,451). These are likely associated with σR stimulation. Recently, a mutation in the σ_1_R has been associated with juvenile ALS (452); therefore, it is not surprising that σ_1_R agonists improve motor function and motor neuron survival in ALS mice (453). In fact, loss of σ_1_R has been associated with defective autophagy and lipid raft disturbances (454).

**Figure 8.**
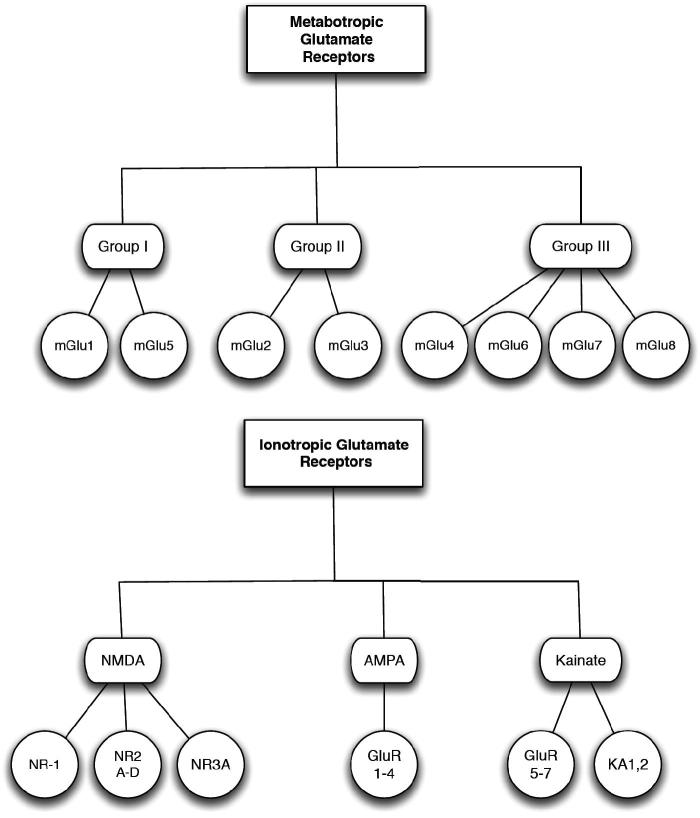
Glutamate receptors types. NMDA – N-methyl-d-aspartate receptor; AMPA – α-amino-3-hydroxy-5-methyl-4-isoxazolepropionic acid; KA – kainate; GluR – glutamate receptor; NR – NMDA receptor subtype.

In contrast to the effects of σR stimulation, antagonism of the σRs blocks compulsive-like eating behavior (455), enhances brain plasticity (456) and exacerbates other addictions (457). In addition, glutamatergic dysfunction has been postulated as being part of the development of disorders associated with long-term plastic changes in the CNS such as chronic pain (458), drug tolerance, dependence, addiction, partial complex seizures and tardive dyskinesia (373,459).

l-Glu acts through both ligand-gated ion channels at the iGluR and at G-protein-coupled metabotropic glutamate receptors [mGluR] (Figure 8). Activation of these receptors is responsible for basal excitatory synaptic transmission and many forms of synaptic plasticity such as LTP and long-term depression [LTD], which are thought to underlie learning and memory (216,371,460–473).

Transporter proteins (Glutamate transporter [GluT]) represent the only significant mechanism for removal of Glu from the extracellular fluid and are important for the long-term maintenance of low and non-toxic concentrations of Glu and appear to have more sophisticated functions in the modulation of neurotransmission (402). A number of soluble compounds, including Glu, cytokines and growth factors, influence the GluT expression and activities (474). It is not known as to whether the σRs are involved in regulation of this transport.

The genes encoding GluT proteins have been cloned both from rats and humans (475–480). They are found in astroglia and microglia widely distributed throughout the CNS (481,482) and provide Glu for synthesis of GABA, glutathione and protein (402,483). They rapidly remove Glu from the synaptic cleft to prevent cell death (484).

Many tissues demonstrate Glu, GluR and GluTs, (396,418,432,434,485–526). mGluRs and l-Glu, l-aspartate and d-aspartate are substrates for the transporters (217,495,521,527), whereas GluR agonists (528) and antagonists (495,529) are not.

GluTs incorporate Glu into cells along with the co-transport of three Na^+^ ions (527,530) and the antiport of one K^+^ ion (529,531) and either one OH^−^ or one 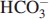 ion. The excess Na^+^ ions generate a net positive inward current, which drives the GluT (527). In addition, a Glu-elicited Cl^−^ current is also associated with some GluT (475,532). In contrast, the vesicular transporter selectively concentrates Glu into synaptic vesicles in a Na^+^-independent, ATP-dependent manner (533–535) that requires Cl^−^ (374,375,533,536–542). Given the complexity of the Glu system and the limited information regarding the interaction of the multiple components with σRs, further research is necessary to fully elucidate interaction of σR and the system components.

##### Glu receptors [GluR]

Two classes of GluRs have been characterized based on studies in the CNS: iGluRs and mGluRs (382,388,389,391,392,421,422,518,543,544). The iGluRs are ligand-gated ion channels that mediate the vast majority of excitatory neurotransmission in the brain. They are classified into three major subtypes: NMDARs, AMPARs and KaRs (296,373,381,382,392–394,449,543,545–554). These receptors exhibit varied pharmacological and electrophysiological properties, including ionic channel selectivity to Na^+^, K^+^ and Ca^2+^ (389,543).

#### NMDA receptors [NMDAR]

The NMDAR is perhaps the best characterized of the iGlu, in part due to the existence of selective agonists and antagonists that can be used to study its physiology. These receptors are modulated by σ_1_R. NMDAR are ubiquitous (555). NMDARs are composed of assemblies of NR_1_ subunits and NR_2_ subunits, which can be one of four separate gene products [NR_2A-D_]. Expression of both subunits is required to form functional channels (556).

NMDARs are structurally complex, with separate binding sites for Glu, Gly, Mg^2+^ (557,558), Zn^2+^ and a polyamine recognition site, where Mg^2+^ ions provide a voltage-dependent block of NMDA-gated channels (394).

NMDAR are highly permeable for Ca^2+^. They show slower gating kinetics with the channel blocked in a voltage- and use-dependent manner by physiological concentrations of Mg^2+^ ions (371,372,458,459,462,559). It is this property of the NMDAR that enables σRs to trigger cell death via Ca^2+^ overload.

#### AMPA receptors [AMPAR]

AMPAR are involved in mediating most forms of fast glutamatergic neurotransmission, which corresponds to a Ca^2+^ influx, a function controlled by the GluR_2_ subunit (560). There are four known subunits GluR_1_ to GluR_4_, sometimes referred to as GluR_A_ to GluR_D_, are widely, but differentially, distributed throughout the CNS (392). AMPARs play an important role in memory function. They are localized in the hippocampus and striatum and also may play a role in the generation of seizures (560–562).

#### Kainate receptors [KaR]

Kainate receptors [KaR] constitute a separate group from the NMDAR and AMPAR, although they share many of the same structural characteristics. KaRs and AMPARs are localized in the hippocampus and striatum and also may play a role in the generation of seizures (563–566). Also they are involved post-synaptically in neurotransmission in some pathways (566–569).

##### Metabotropic Glu receptors [mGluR]

mGluRs form a family of currently eight subtypes (mGluR_1–8_), subdivided into three groups (I–III) (570–572). Activation of group-II (mGluR_2,3_) or group-III mGluRs (mGluR_4,6–8_) has been established to be neuroprotective *in vitro* and *in vivo* (572), and for the NMDA iGluR (573). In contrast, group-I mGluRs (mGluR_1,5_) need to be antagonized in order to evoke protection (448) antagonists, and drugs acting on 5-HT_2A,_ α_2_-adrenergic, adenosine (A2A) and cannabinoid [CB_1_] receptors may be helpful (574).

Members of this family of mGluR exert their effects either on the second messengers or ion channels via the activation of the GTP-binding proteins and regulate the synthesis of different intracellular second messengers such as IP_3_, cAMP or cGMP, as do σRs (382,422). They function to modulate the presynaptic release of Glu and the post-synaptic sensitivity of the cell to Glu excitation (382,389,390,392,422).

mGluRs have both chemical and electrical signaling properties (575). Glu binding onto an mGluR opens non-selective cation channels more permeable to Na^+^ and K^+^ ions than Ca^2+^ (548,576). mGlu binding elicits a rapidly activating inward and outward current and Ka, quisqualate and AMPA are the specific agonists at these receptors (399,577–583).

As with iGluRs, the mGluRs are classified into 4 groups (Group I–IV) based on AA sequence similarities, agonist pharmacology and the signal transduction pathways to which they are coupled (584). Each receptor is formed from the co-assembly of several subunits (584–587). To date, eight subunits (named GluR_1_ through GluR_8_) have been cloned (393,576,586,588–591).

##### σRs and Glu neurotransmission

Numerous studies have shown interactions between σRs and NMDAR-mediated responses. For example, σR ligands, including haloperidol, (+)-PTZ, 4-IBP (592), (+)-3-PPP, (+)-SKF-10,047 (593) and DTG (594), antagonize NMDAR currents in *Xenopus* oocytes (595). The effects of σR ligands on NMDARs in are thought to be indirect; however, high doses (µM) and nonselective σR ligands have been used in past studies. Furthermore, there was no correlation between the potency of NMDAR inhibition and the affinity or stereo selectivity for σR sites (595–597). Thus, it is difficult to assess whether these observations have been based on σR mediated actions rather than on non-specific effects. *In vitro* radioligand binding studies have shown that haloperidol, (+)-PTZ, DTG, (+)-SKF-10,047 and (+)-3-PPP inhibited [3H]TCP binding to NMDARs in neuronal cells, with a potency correlated with the affinity for DTG binding sites (64,598).

In a model of modulation of the NMDA response in dorsal hippocampal pyramidal neurons of the CA_3_ region, it was found that low doses of the σR ligands DTG, JO-1784, (+)-PTZ and l-687,384 selectively potentiated the response of these neurons to microiontophoretic applications of NMDA (137,194,195,197). Other σR ligands such as BD-737, 4-IBP and OPC-14523 were less selective (196,376).

Interestingly, it was also found that depending on the initial level of excitatory response to QUIS and NMDA, σR agonists could increase or decrease NMDA-induced responses, thus suggesting a real modulatory role of σR ligands on the Glu response (377). Antagonists including SA4503 (593), BMY-14802, (+)-3-PPP and NE-100, suppress the potentiation induced by σR agonists (162,195,197).

The effects of all σ_1_R agonists on the NMDA response produce a biphasic dose response curve, which will be discussed later (194,376,377). As stated above, this particular pharmacological profile could explain the discrepancies observed for the effects of σR ligands with respect to inhibition versus potentiation on NMDAR-mediated responses, as most *in vitro* studies may have used high doses, at which the σR ligands were acting as antagonists.

In contrast, the antidepressants paroxetine and tranylcypromine, which have a low affinity for σRs, have no effect on the NMDA response despite their similar monoaminergic profiles to sertraline and clorgyline. Moreover, the effects of sertraline and clorgyline are suppressed by the σR antagonist haloperidol but not by spiperone, suggesting that their effects are likely mediated by σRs (197). The σ_2_R ligands Lu 28-179 (599) and BD-1008 (600) have also been shown to modulate NMDA mediated responses.

Despite their high affinity for σ_2_Rs, the doses required for antidepressant activity are 5–10 times higher than σ_1_R ligands (102). The effects of the specific σ_2_R ligand Lu 28–179, are not blocked by the σ_1_R antagonists NE-100, PROG, or haloperidol, suggesting that these effects are mediated through σ_2_R (102).

*In vitro* models have also suggested a modulatory role for σR agonists on NMDA-mediated responses. For example, JO-1784, BD-737, (+)-PTZ and (+)-3-PPP potentiates in a concentration-dependent manner NMDA-induced [^3^H]NE release from preloaded rat hippocampal slices (135,162,210), whereas DTG and BD-737 act as inverse agonists, by concentration dependently inhibiting the overflow of [^3^H]NE evoked by NMDA. Haloperidol and BD-1063 (208) alone do not modify [^3^H]NE release, but completely prevent the effects of JO-1784, BD-737, (+)-PTZ, DTG and (+)-3-PPP (162), whereas DuP734 inhibits that of BD-737 (122).

Neurosteroids, acting as σR agonists, have also been shown to modulate NMDAR-mediated effects (601), as DHEA at low doses potentiates the NMDA response in extracellular recordings from the dorsal hippocampus. The effect of DHEA is blocked by NE-100 and haloperidol (123,179). In this model, neither PREG nor PREGS modifies the NMDA response or act as antagonists (602), which may be due to their lower affinity for the σ_1_R (82,283).

Endogenous hormone levels also affect the σRs modulatory effect on NMDA-mediated responses. For example, two weeks following OVX, the potentiation of the NMDA response induced by DTG was significantly greater than in control female rats, suggesting that σRs may be tonically inhibited by endogenous PROG (123,180). In agreement, 10 times higher doses of (+)-PTZ and DHEA are required in pregnant females to potentiate the NMDA response. This reduction of effect of σR agonists in late pregnancy may be due to occupation of σR by high concentrations of PROG and the apparent super sensitivity of σR observed during the post-partum period that might be due to the rapid drop of PROG levels after parturition (603–605). Overall, many σ_1_R ligands have demonstrated the ability to modulate NMDA-mediated Glu neurotransmission.

#### γ-Aminobutyric acid (GABA)

Glutamic acid decarboxylase [GAD] in mouse brain is capable of decarboxylating Glu to GABA but requires pyridoxal 5-phosphate as a cofactor (606–611). The role of GABA as a neurotransmitter is that of inhibitory neurotransmission, although this property has been questioned recently (612). Following the purification of GAD and the generation of GAD antisera, immunohistochemical studies reveal that many GABAergic neurons in brain are interneurons and are, therefore, uniquely able to alter the excitability of local circuits within a given brain region (611,613). From these and other studies it has been confirmed that 30–40% of all CNS neurons utilize GABA as their primary neurotransmitter.

GABA is formed *in vivo* via a metabolic pathway called the “GABA shunt.” The initial step in this pathway utilizes α-ketoglutarate formed from glucose metabolism via the Krebs cycle. α-Ketoglutarate is then transaminated by α-oxoglutarate transaminase (GABA-T) to form Glu, the immediate precursor of GABA. Finally, Glu is decarboxylated to form GABA by the GAD (607,608,614). GAD is expressed only in GABAergic neurons and in certain peripheral tissues, which are also known to synthesize GABA (615).

The principal neuronal GABA transporter is a 70–80 kDa glycoprotein that contains 12 hydrophobic membrane-spanning domains (616,617). Specific inhibitors of GABA uptake that directly bind to the transporter have anticonvulsant and antinociceptive properties in laboratory animals (500). The role of σR interaction with GABA and GABA transporters has yet to be elucidated, but given their role in NMDARs, a role for them could be postulated.

Conformationally-restricted analogues of GABA have been used to help identify three major GABARs, termed GABA_A_(618–620), GABA_B_ and GABA_C_ receptors (621,622) GABA_A_ and GABA_C_ receptors are members of a superfamily of transmitter-gated ion channels that include nACh (623), strychnine-sensitive Gly and 5-HT_3_ receptors (618,619). On the other hand, GABA_B_Rs are seven transmembrane receptors that are coupled to G-proteins and activate second messenger systems and Ca^2+^ and K^+^ ion channels, resembling the activity of mGluRs (624).

The large numbers of drug recognition sites associated with GABA_A_Rs, suggested that there may be an endogenous receptor ligand including two natural reduced steroid metabolites of PROG and deoxycorticosterone: ALLO and allotetrahydro-DOC (619,625,626). However there is little compelling evidence at present that any interact with GABA_A_Rs *in vivo* (627). More recently, N,N-dimethyltryptamine [DMT] has been shown to be the endogenous ligand of σRs (7,8), not the neurosteroids as previously thought.

To date, five distinct classes of polypeptide subunits (α, β, γ, δ and ρ) have been cloned (618,620) and multiple isoforms of each have been shown to (628). There is approximately 70% sequence identity between the polypeptide subunits within a given class, but only approximately 30% between classes (629,630–632).

#### Glycine [Gly]

Gly is the major inhibitory neurotransmitter in the brainstem and spinal cord and functions as a co-agonist at the NMDA subtype of GluR, finely modulated by local expression of specific Gly transporters such as GLYT_1_ (633) in the forebrain, where it promotes the actions of Glu, the major excitatory neurotransmitter (449). Thus, Gly serves both inhibitory and excitatory functions within the CNS.

The actions of Gly are terminated primarily by reuptake via Na^+^–Cl^−^-dependent, high-affinity Gly transporters [GlyT]. Like GABA, this increase in Cl^−^ ion conductance results in a hyperpolarization of the neuronal membrane and an antagonism of other depolarizing stimuli (634). Given their impact on NMDARs, σ_1_Rs and their activation are probably potentiating factors for glycine transmission.

#### Cannabinoids

Cannabis and cannabinoids exert most of their biological functions through receptor-mediated mechanisms. Two types of cannabinoid receptors [CB] have been identified – namely CB_1_ and CB_2_ – both coupled to a G protein (635). CB_1_ receptors have been detected and quantified in the CNS (636). They are responsible for the characteristic effects of cannabis, including catalepsy, depression of motor activity, analgesia and feelings of relaxation/well being. Cannabis also affects peripheral neurons; activation of CBs produces suppression in neurotransmitter release in the heart, bladder, intestine and *vas deferens* (637,638).

CB_1_ cannabinoid receptors appear to mediate most, if not all of the psychoactive effects of δ-9-tetrahydrocannabinol [δTHC] and related cannabinoid compounds. This G protein-coupled receptor has a characteristic distribution in the nervous system: It is particularly enriched in cortex, hippocampus, amygdala, basal ganglia outflow tracts and cerebellum, a distribution that corresponds to the most prominent behavioral effects of cannabis (637).

Cannabinoid CB_2_ receptors have only been detected outside the central nervous system, mostly in cells of the immune system, presumably mediating cannabinoid-induced immunosuppression and anti-inflammatory effects (639). With the discovery of cannabinoid receptors for exogenous cannabinoids, endogenous cannabinoids (anandamide, 2-arachidonylglycerol [2-AG]) have been described subsequently (638,640).

Endocannabinoids not only act at cannabinoid receptors, but potentially also at vanilloid and 5-HT_3_ receptors, both of which are expressed in the gastrointestinal tract. The interactions between endocannabinoids and these other important receptor systems have not been extensively investigated (641). Additionally, experimental evidence also suggests that endocannabinoids mediate neuron-astrocyte communication (642).

The relationship of cannabinoids to the σRs has received little attention, although the interaction of CB_1_ with the classical opiate receptors has been investigated (635). Decades ago, it was been shown that the morphine-induced dopamine release in the nucleus accumbens requires the CB_1_ activation (643). Although studies seldom include investigation of the σRs, the effects on other neurotransmitter systems suggest a possibility of interaction of the CB and σRs. For example, both the serotonergic (644) and endocannabinoid systems modulate frontocortical Glu release (645). Cannabinoid CB_1_ receptor antagonists rimonabant (SR141716) and AM251 directly potentiate GABA_A_ receptors (646), inferring that CB_1_ receptor agonists may do the reverse; thus damping the excitatory effects of Glu. In fact, endocannabinoids control GABA effects (647), mediates inhibition Glu transmission in the hippocampus (648) leading to the neuroprotective role on the cannabinoids (649) via negative signaling through the G-protein-coupled cannabinoid receptors (650). Although specific, direct data are absent for the role that σRs play in the cannabinoid modulation, the role that σRs play in Glu modulation suggests that they are probably involved in the modulation of Glu produced by the endocannabinoids. To support this hypothesis, the endocannabinoid system plays a central role in the phenomenon of addiction (651), as do the σRs. Hence, some of the changes in σR signaling seen in addictions probably occur in concert with the endocannabinoid system. In fact, it has recently been suggested that σ_1_R dysfunction might increase vulnerability to cannabis-induced psychosis (652).

#### Summary

Summarizing the interactions of σR with neurotransmitters is difficult. Data are scarce and incomplete. In addition, the dose-response of stimulation of the σR to an agonist can show stimulatory effects at a low dose and inhibitory effects at high doses, when used experimentally using greater concentrations than physiological levels. As most work is done in *in vitro*, doses are often excessive and may reflect an overexposure that would not be seen in the *in vivo* situation. Nonetheless, it seems clear that the σRs have a core and only partly defined role in regulation of neurotransmission.

## Pharmacology

A diverse class of psychotropic drugs bind to σ_1_Rs (653), including antipsychotics, e.g. haloperidol [Haldol®], which have the highest affinity for σ_1_R (176,177,654), SSRIs, which have medium to high affinities for σ_1_Rs, and tricyclic antidepressants [TCAs], which have less (176,655). Other compounds that bind to the receptor include morphinans (e.g. DEX) (69), guanidines (e.g. DTG) (193), phenothiazines (e.g. chlorpromazine) (656), butyrophenones (e.g. haloperidol) (657), TCAs (e.g. imipramine) (658), monoamine oxidase inhibitors [MAOI] (e.g. clorgyline) (659), SSRIs (e.g. sertraline) (660), cytochrome P_450_ inhibitors (e.g. proadifen) (192), anticonvulsants (e.g. phenytoin) (141), addictive drugs (e.g. cocaine, METH) (661), polyamines (e.g. ifenprodil) (662) and certain steroids (e.g. progesterone [PROG] and testosterone) (98). The effects of cocaine occur through direct involvement of σ_1_R and the DA_1_ receptor (663). In addition, the anticonvulsant drug phenytoin allosterically modulates σ_1_Rs (141). These receptors also exhibit a high affinity for (+)-isomers and are proposed to be associated with both pertussis toxin-sensitive G*_i_*_/o_ and cholera toxin-sensitive G_s_ proteins, PLC and PKC (165).

The σ_1_Rs have an affinity for a number of specific stereoisomers of these drugs (e.g. (+) PTZ and (+) cyclazocine) (65,79,179,252,653,664). The lack of selectivity between the σ and PCP binding sites seen following exposure to several compounds, including BZM or PCP derivatives, led to a confusion resolved by the availability of new highly selective drugs. Among them, the reference PCP non-competitive antagonist (+)MK-801 maleate [dizocilpine] failed to displace radioligands labeling the σR sites. Selective σR agonists like 1,3-di-O-tolylguanidine [DTG], (+)N-cyclopropylmethyl-N-methyl-1,4-diphenyl-1-ethyl-but-3-en-1-ylamine hydrochloride [JO-1784, igmesine], 2-(4-morpholino)ethyl-1-phenylcyclohexane-1-carboxylate hydrochloride [PRE-084] and 1-(3,4-dimethoxyphenethyl)-4-(3-phenylpropyl)piperazine dihydrochloride [SA4503], do not bind to the NMDAR-associated PCP site (212). These compounds are now reference compounds in terms of selectivity between σ and PCP receptors.

Neurochemical and electrophysiological studies have then been crucial in revealing the function of the σR (26,129,130). These studies have demonstrated that σRs play a role as modulators of Ca^2+^ release (164,665) and inhibitors of voltage-gated potassium K^+^ channels (138,139), NMDARs, tyrosine kinase [TK]-related processes (666), I*P_3_*R activation (139,161,164,165), other iGluR and mGluR functions (548), neurosteroids (667) and other neurotransmitter activities (211).

σ_1_Rs also regulate compartmentalization of lipids in the ER (64,65,164,668,669), and have antitumor activity *in vitro* and *in vivo* (670). Studies also have suggested that σ_1_Rs regulate lipid transport and metabolism, neurogenesis (671), cellular differentiation and myelination in the brain (672); the latter has implications for diseases such as multiple sclerosis [MS].

The actions mediated by σ_1_Rs at the cellular level can be considered either as acute or chronic. The acute actions include the modulation of ion channels (e.g. K^+^ channel), NMDARs, IP_3_R and σ_1_R translocation. Chronic actions of σ_1_Rs are basically considered to be the result of an up- or down regulation of the σ_1_R itself. For example, the up regulation of σ_1_R *per se*, even without exogenous ligands, promotes cellular differentiation and reconstitution of lipid “micro domains” in cultured cells (65,673). Recent *in vitro* and *in vivo* studies strongly point to the possibility that σ_1_Rs participate in membrane remodeling and cellular differentiation in the nervous system (65).

Metabolic studies support the view that σRs have functional significance in brain glucose metabolism as glucose utilization is affected by ligands in areas of brain that show high densities of σRs (78). The findings of up and down regulation, suggest that σ_1_Rs might possess a constitutive biological activity, and that σ_1_R ligands might merely work as modulators of the innate activity of this protein.

σ_1_Rs are present throughout vertebrate evolution, with conserved pharmacologic properties (674), in sea anemones, planaria, earthworm, crayfish, cricket, hadfish, shark, goldfish, frog, turtle, chicken, guinea pig and monkey. There does not appear to be a family-related trend in quantity, e.g. monkey has only 20% of the σRs seen in the guinea pig (675). Also, σRs differ from classical neurotransmitter receptors in that they show no postnatal ontogeny in the rat and no age-dependent change in the receptor density. The lack of postnatal development of receptors in the CNS, as compared with postnatal changes in other classical neurotransmitter receptors, and the fact that σR sites are much denser in peripheral organs, such as the liver (418), immune and endocrine tissues (676, 677), suggest a universal role for σRs in cellular function. Because of their widespread modulatory role, σ_1_R ligands have been proposed to be useful in several therapeutic fields such as amnesic and cognitive deficits, depression and anxiety, schizophrenia, analgesia and against some effects of drugs of abuse (such as cocaine and METH) (32,678).

### **σ**_1_R ligands

σR agonists and antagonists are common in easily available drugs, e.g. DEX in cough medications. These antagonists show a GTP-sensitive high affinity binding to the σ_1_R (679). A complete list of σR ligands is difficult to obtain, as many compounds only have been used in research (680) and are not available, due to the proprietary nature of drug development. A short list of σ_1_R and σ_2_R ligands can be seen in Table 1.

**Table 1.** Some σR ligands in order of their potency.

σR ligands in rough order of potency	
Sigma_1_ ligands	Sigma_2_ ligands
(+)-pentazocine [PTZ]	1,3 di-o-tolyl-guanidine [DTG]
Haloperidol [Haldol®]	Haloperidol [Haldol®]
1,3di-o-tolyl-guanidine [DTG]	(+)-3-PPP [preclamol]
(+)-3-PPP	(−)-pentazocine [PTZ]
Dextromethorphan [DEX]	Phencyclidine
(+)-SKF 10,047	(+)-pentazocine [PTZ]
(+)-cyclazocine	(−)-SKF 10,047
(−)-pentazocine [PTZ]	BD1047
Phencyclidine	BD1063
(−)-SKF 10,047	

Each of the above acts as either an agonist or antagonists often depending on the dose. This biphasic dose-response makes evaluation of studies difficult, but does help to explain conflicting findings (681). Bearing in mind the biphasic dose-response of agonists and antagonists, a summary of some ligands and their agonistic or antagonistic activities can be seen in Table 2.

**Table 2.** σR agonists and antagonists.

σ_1_R and σ_2_R ligands as agonists or antagonists	
σR ligands – agonists	σR ligands – antagonists
(+)-N-allylnormetazocine [(+)-SKF 10,047]	(1-[2-(3,4-dichlorophenyl)ethyl]-methylpiperazine [BD1063]
2-(4-morpholino)ethyl-1-phenylcyclohexane-1-carboxylate [PRE-084]	(N-(3,4-dichloropheny)ethyl]—4-methylpiperazine [BD 1008]
1-(3,4-dimethoxyphenethyl)-4-(3-phenylpropyl)piperazine [SA 4503]	[1-cyclopropylmethyl)-4-(2′(4″-flurophenyl)-2′-oxoethyl)piperidine [DuP 734]
1′-[4-[1-(4-fluorophenyl)-1H-indol-3-yl]-1-butyl]- spiro[isobenzofuran-1(3H), 4′piperidine] [Lu 28-179]	2-amino-7phosphonoheptanoic acid [AP-7]
Fluoxamine	NE-100
Pregenolone-S	E-5842
DHEA-S	BD1139
Donepezil	BIMU-8
PPBP	BMY 14802
Amitriptyline	Cabetapentane
BD 737	Dextromethorphan [DEX]
Ibogane	Eliprodil [SL 82.0715]
Haloperidol (σ_2_R) [Haldol®]	Fenpropimorph
BD 737	Haloperidol (σ_1_R) [Haldol®]
4-(N-benzylpiperidin-4-yl)-4-iodobenzamide [4-IBP]	Ifenprodil tartrate
3,4-methylenedioxymethamphetamine [MDMA]	N,N-dipropyl-2-[4-mrthoxy-3-(2-phenylethoxy)phenyl]ethylamine monohydrochloride [NE-100])
Dehyroepiandrosterone sulfate [DHEA-S] [suggested as the endogenous σR agonist]	N-2-[2-(3,4-dichlorophenyl)ethyl]-N-methyl-2-(dimethylamino) ethylamine [BD 1047]
(+)Cyclozocine	N-methyl-d-aspartate [NMDA]
Siramesine	N-phenthylpiperidine
Igmesine	Opipramole
Fluvoxamine	Panamasine
Dextromethorphan [DEX]	Pregnalone
OPC-14523	PROG (suggested as the endogenous σR antagonist)
CB-64D	Rimcazole
Ditolylguanidine [1,3-di-O-tolyguanidin] [DTG]	Sabeluzole
Memantine	Testosterone
Certain steroids (agonist plus steroidal effect)	Tiopirone
Phencyclidine [PCP]	Verapamil
Donepezil	WAY 100635
Igmesine [J01783]	Sertraline
Interleukin 10 [I l-10]	
(+)3H-3-3(3-Hydroxyphenyl)-N-(1-propyl)-piperidine [SA4503]	
Methamphetamine	
Phenothazines	
(+)-pentazocine [PTZ]	
Heroin	
Cocaine	
2-(4-morholinethyl)1-phenycyclohexanecarboxylate	
Amantadine	
CB-184	
Dimemorfan	

### Dose response

As previously mentioned, σRs do not show a linear dose-response curve, but show a biphasic dose response curve in various behavioral, biochemical and electrophysiological paradigms (135,137,161,182,194,195). For example, the σR agonist, SA4503, both attenuates and enhances the effects of methamphetamine depending on the dose (682).

A similar dose response curve has also been described with σR ligands in other models such as in release experiments (135) and in behavioral models (182,184). It has been proposed that the different dose response may be due to low doses of σR ligands activating one subtype of σRs for which they have high affinity, whereas higher doses may activate another subtype(s) of the σR for which they have a lower affinity). Such activity would counteract the effects observed at lower doses (194,196,197). Nonetheless, it is important to note that the different, and sometimes opposite, results obtained with low and high doses of σR ligands may explain much of the controversy seen in the literature on σRs.

The fact that low dose effects do not follow the classical dose-response curve has been known for many years and has only been recently revived under the title of hormesis (683–709). The *Arndt-Schulz rule* or *Schulz’ “law”* is a basically a hypothesis concerning the observed effects of many chemicals in low concentrations (710–713). According to the Arndt-Schulz rule, highly diluted chemicals enhance life processes, while strong concentrations of the same chemical may inhibit these processes and even terminate these processes (714).

Depending on the process affected, this interplay results in either a J-shaped or inverted J-shaped dose response, which are sometimes called “bell-shaped”, “U-shaped,” “inverted U-shaped,” “biphasic” or “β-curve” (685,686,710,715–732). The point at which the hormetic curve crosses the reference level of response (i.e. the threshold) is the zero equivalent point [ZEP]; in other words, the point at which there is no toxic or stimulatory effect.

At low doses of an σR agonist induction and potentiation of the NMDA response is seen (162,195). In contrast, at higher doses the effects of σR agonists such as DEX, and Igmesine (733) progressively decrease and disappear (194). A similar dose response curve has also been described with σR ligands in other models such as in neurotransmitter release experiments (135) and in behavioral models (182).

It has been proposed that the biphasic dose response curves may be explained by low doses of an σR ligand activating one subtype of σR for which they have high affinity, whereas higher doses activate another or other subtype(s) of the σRs for which they have a lower affinity.

Such a mechanism would counteract the effects observed at lower doses (194,196,197). Nonetheless, it is important to note that the different, and sometimes opposite, results obtained with low and high doses of σR ligands.

### Models

σR ligands have been proposed for tumor imaging studies (734), particularly in the detection of pulmonary and abdominal tumors (735), despite irreversible binding in some cases, e.g. (^11^C)-SA5845 (736). In fact, selective σ_2_R ligands preferentially bind to pancreatic adenocarcinomas; thus, expanding the possibility of σ_2_R-based applications in diagnostic imaging, in addition to therapy (110) or drug development (239). A haloperidol challenge has shown that [^123^I]TPCNE is a novel is a single photon emission-coupled tomography [SPECT] tracer for the σ_1_R (737).

A σ_1_R knockout mouse has been developed. The mice demonstrated no overt abnormal phenotype when compared to the wild type. The activity of σ_2_Rs seems to be unaffected in σ_1_R-mutant mice. (63). As expected, however, they do lack the locomotor response to the σR ligand (+)-SKF100047 and display reduced response to pain via the σ_1_R (39).

### Cell development and plasticity

σR drugs and neurosteroids, acting at the level of the σ_1_R protein, are important for plasticity, cell development and trophic actions. These are probably mediated by Ca^2+^ (82,185,190,210). This observed plasticity, which is both associated with the same prerequisite enhancement of NMDA-mediated glutamatergic neurotransmission and protein dephosphorylation that occur downstream from the massive entry of Ca^2+^, and [Ca^2+^]*_i_* mobilization from the endoplasmic reticulum and the mitochondria, often occur synergistically (188,738).

Intracellular chaperones, reside specifically at the endoplasmic reticulum (ER)-mitochondrial interface, referred to as the mitochondrial-associated ER membrane [MAM]. Here, σ_1_Rs is an inter-organelle signaling moderator (665) and regulates ER-mitochondrion Ca^2+^ signaling (739).

As previously mentioned (88,133,139,145,146,740), K^+^ channels, which control the fine tuning of Ca^2+^ entry through both VSCCs and SOCs (store-operated channels), are also prominent targets of the σ_1_R agonist and antagonist drugs.

### Age changes in σRs

Throughout adulthood, differences in the motor changes elicited by drugs affecting σR are correlated with the number of receptors in the P_2_, and not the P_3_, cellular fraction (198). Thus, translocation of the σ_1_R from the ER to the cell membrane (190) decreases with age in motor neuron regions. An increase in density of σ_1_Rs found in the aged monkey brain supports this hypothesis (199), as they are not as readily translocated and, therefore, increase in density.

For this reason it has been suggested that age-related memory deficits associated with advancing age may be responsive to up regulation of the σRs. In fact, such ability to alleviate memory deficits during aging has been confirmed in humans for the selective σ_1_R agonist (+)-*N*-cyclopropylmethyl-*N*-methyl-1,4-diphenyl-1-ethyl-but-3-en-1-ylamine hydrochloride [Igmesine®], which appears more efficient among the elderly (206).

Conversely, the σ_2_R subtype exhibits no stereo selectivity and only low affinities for the (+)-BZM. It does not appear to be modulated by pertussis toxin-sensitive G*_i_*_/o_ proteins (207), and is predominantly located in the motor system and periphery (21). Interestingly, brainstem motor function, which is profoundly sensitive to σR drugs, decreases with age, resulting in the reduced accuracy and consistency of fine and complex motor performance (208).

In addition to other neurosteroids, which change with age, the discussion above reflects the effects of DHEA(S) on σ_1_Rs. Therefore, the age-related changes in neurosteroids probably affect the σRs. Alteration of age-related changes in memory probably relates to the balance of excitatory and inhibitory effects on the CNS, in which σRs play a role. Several studies in rodents show that GABA_A_ agonists impair learning and memory while GABA_A_ antagonists enhance memory (213,214).

In humans benzodiazepines [BZD] (σR agonist) may impair cognition (741–743). On the other hand, σR agonists enhance memory performance in young rodents and in rodent models of cognitive impairment (182,200,201,203,744). In addition, the NMDAR is involved in the development of long-term potentiation [LTP] (215,216), an essential element of neural plasticity. In addition, it now appears as though DHEA(S) has an ability to modulate neurotransmitter receptors in the CNS that are primarily involved in learning and memory (209). Thus, σRs appear to be essential for maintaining neural health and protecting against age-related mammary defects.

### Sigma ligands

Numerous σR agonists and antagonists have been previously described. Some are common and easily available drugs, e.g. DEX in cough medications. A short list of σ_1_R and σ_2_R ligands can be seen in Table 3.

**Table 3.** Some σR ligands in order of their potency.

σR ligands in rough order of potency	
σ_1_R ligands	σ_2_R ligands
(+)-pentazocine [PTZ]	1,3 di-o-tolyl-guanidine [DTG]
Haloperidol	Haloperidol
1,3 di-o-tolyl-guanidine [DTG]	(+)-3-PPP [preclamol)
(+)-3-PPP	(−)-pentazocine
Dextromethorphan [DEX]	Phencyclidine
(+)-SKF 10,047	(+)-pentazocine
(+)-cyclazocine	(−)-SKF 10,047
(−)-pentazocine	BD1047
Phencyclidine	BD1063
(−)-SKF 10,047	

Each of the above acts as either an agonist or antagonists. A list of agonists and antagonists can be seen in Table 4.

**Table 4.** σR agonists and antagonists.

σ_1_R and σ_2_R ligands as agonists or antagonists	
σR ligands – agonists	σR ligands antagonists
(+)-N-allylnormetazocine [(+)-SKF 10,047]	(1-[2-(3,4-dichlorophenyl)ethyl]-methylpiperazine [BD1063]
2-(4-morpholino)ethyl-1-phenylcyclohexane-1-carboxylate [PRE-084]	(N-(3,4-dichloropheny)ethyl]—4-methylpiperazine [BD 1008]
1-(3,4-dimethoxyphenethyl)-4-(3-phenylpropyl)piperazine [SA 4503]	[1-cyclopropylmethyl)-4-(2′(4″-flurophenyl)-2′-oxoethyl)piperidine [DuP 734]
1′-[4-[1-(4-fluorophenyl)-1H-indol-3-yl]-1-butyl]-spiro[isobenzofuran-1(3H), 4′piperidine] [Lu 28-179]	2-amino-7phosphonoheptanoic acid [AP-7]
Amitriptyline	E-5842
BD 737	BD1139
Ibogane	BIMU-8
Haloperidol (σ_2_R)	BMY 14802
BD 737	Cabetapentane
4-(N-benzylpiperidin-4-yl)-4-iodobenzamide [4-IBP]	Dextromethorphan [DEX]
3,4-methylenedioxymethamphetamine [MDMA]	Eliprodil [SL 82.0715]
Dehyroepiandrosterone sulfate [DHEA-S] [suggested as the endogenous σR agonist]	Fenpropimorph
(+)Cyclozocine	Haloperidol (σ_1_R)
Siramesine	Ifenprodil tartrate
Igmesine	N,N-dipropyl-2-[4-mrthoxy-3-(2-phenylethoxy)phenyl] ethylamine monohydrochloride [NE-100])
Fluvoxamine	N-2-[2-(3,4-dichlorophenyl)ethyl]-N-methyl-2- (dimethylamino)ethylamine [BD 1047]
Dextromethorphan [DEX]	N-methyl-d-aspartate [NMDA]
OPC-14523	N-phenthylpiperidine
CB-64D	Opipramole
Ditolylguanidine [1,3-di-O-tolyguanidin] [DTG]	Panamasine
Memantine	Pregnalone
Certain steroids (agonist plus steroidal effect)	PROG (suggested as the endogenous σR antagonist)
Phencyclidine [PCP]	Rimcazole
Donepezil	Sabeluzole
Igmesine [J01783]	Testosterone
Interleukin 10 [I l-10]	Tiopirone
(+)3H-3-3(3-Hydroxyphenyl)-N-(1-propyl)-piperidine [SA4503]	Verapamil
Methamphetamine	WAY 100635
Phenothazines	
(+)-pentazocine	
Heroin	
Cocaine	
2-(4-morholinethyl)1-phenycyclohexanecarboxylate	
Amantadine	
CB-184	
3-(4-(4-cyclohexylpiperazin-1-yl)butyl)benzo[d]thiazole-2(3H)-thione (CM156)	
Dimemorfan	

## Known σRs – location and effects

Steroids binding to σRs has suggested that σRs serve as a link among endocrine, nervous, heart, lung, kidney, liver, intestines, and sexual (745) and immune systems (221); hence, evaluation of σRs in one organ in isolation can miss a significant lesion that is only apparent when viewed in conjunction with other σR-containing organs showing similar, but subtler, lesions.

The tissue density of σRs is not uniform and is different for each subtype (376). The highest concentration of receptors is seen in the CNS, followed by the periphery (liver, spleen, endocrine, GIT lung) (171,676,746–749).

Links between σ_1_Rs and G-proteins and mGlu (133,138,139,142,750) implies that the σ_1_R mediates a large number of its effects via the Glu system and as such Glu-related diseases probably have an σR component to them.

The σ_1_R has been implicated in myriad of disease phenomena, including cardiovascular arrhythmias (751,167), schizophrenia (15), clinical depression [DEP] (752), Parkinsons disease [PD] (574), Alzheimers disease [AD] (753), the effects of cocaine abuse (754) and cancer (95,377,755,756). σ_1_Rs are distributed throughout the brain in normal subjects, but decreased in the frontal, temporal and occipital lobes, cerebellum and thalamus in patients with early AD and in the putamen in patients with PD (757). Compromising σ_1_Rs at the endoplasmic reticulum results in cytotoxicity in a dose response manner at physiologically relevant concentrations of dopamine (758). In fact, the cytotoxicity of σR agonists is associated with major changes in cellular metabolism when there is occupancy of the σ_2_R (759). More recently, the pharmacological stimulation of the σ_1_R has shown some neuro-restorative effects in experimental PD (760).

### Central nervous system

As previously outlined, the CNS appears to be the primary site of σR activity and effects. Specific regions that have been shown to have concentrations of σ_1_R include, but are not limited to, corpus striatum, nucleus accumbens (61), *substantia nigra, pars compacta* (656), hippocampal pyramidal cell layer (761), hypothalamus, central grey and red nucleus, pontine and cranial nerve nuclei, pontine nuclei, pons – medulla (761), amygdala (762) and cerebellum (763). σ_1_Rs have also been seen in the spinal cord, particularly the ventral and dorsal route ganglia (761), a site that is important for σR agonist induction of neck dystonia in rats (764). More specifically, the regional distribution of σR binding within the brain has shown densities at sites as follows: medulla-pons > midbrain > cerebellum > thalamus > striatum > cortex > hippocampus (78,79).

Studies comparing σ_1_R versus σ_2_R distributions have established that σ_1_R sites are most abundant in the dentate gyrus of the hippocampus, facial nucleus, thalamic and hypothalamic nuclei, with moderate densities found in the striatum, cerebellum, dorsal raphe nucleus and locus coeruleus (171,302,765,766). In agreement, studies of σ_1_R mRNA levels have found high levels of expression in all layers of the cerebral cortex, striatum, hippocampus and cerebellum (767). In comparison, σ_2_R sites are prominent in the *substantia nigra*, central gray matter, oculomotor nuclei, cerebellum, *nucleus accumbens*, amygdala, olfactory bulb, hippocampus and motor cortex (766,768).

The σRs are probably essential for Glu regulation. Glu neurons, also containing GluRs, make up an extensive network throughout the cortex, hippocampus, striatum, thalamus, hypothalamus, cerebellum, and visual and auditory centers in the CNS (386,417,769,770).

At the cellular level, in the CNS, the σ_1_R is expressed in neurons, ependymocytes, oligodendrocytes and in the peripheral nervous system [PNS], Schwann cells (171,672,771–773). GluRs are similarly expressed in these regions (485), which may mediate the excitatory influence of the σRs.

The highest levels of σ_1_R immunostaining can be observed in the neurons of the granular layer of the olfactory bulb, hypothalamic nuclei and pyramidal layers of the hippocampus (171). Among other areas that exhibit intense to moderate σ_1_R immunostaining are the superficial cortical layers, striatal areas including the CP and nucleus accumbens (core and shell), the midbrain, the motor nuclei of the hindbrain, cerebellar Purkinje cells in the cerebellum and the dorsal horn of the spinal cord. At the subcellular level, the σ_1_R is mostly present within neuronal perikarya and dendrites, where it is associated with microsomal, plasmic, nuclear, or ER membranes (171).

There is adequate direct and circumstantial evidence for abnormal Glu and Glu analogue neurotransmission, suggesting altered σR activity, in the etiology and pathophysiology of many neurological and psychiatric disorders such as epilepsy, schizophrenia, addiction, DEP, anxiety, AD, HD, PD and ALS. In fact, it has been suggested that the lack, or dysfunction, of σ_1_R exacerbates ALS (774) and AD (42).

σRs probably dampen the excitotoxic effect Glu. Excessive Glu effects can be pronounced during acute events such as ischemic stroke and trauma, or milder but prolonged in chronic neurodegenerative diseases such as AD, PD, HD and ALS (425,447,450,451,775,). In addition there appears to be a role for Glu system, and hence σRs, in regulation of manganese [Mn^2+^], mercury [Hg^2+^] and lead [Pb^2+^] neurotoxicity (379) (Table 5).

**Table 5.** Summary of σ_1_R and associated psychiatric diseases.

Disorder	Substances tested	Study type	Events (121)
Schizophrenia	Haloperidol	Nonclinical	σ_1_R ligands modulate NMDA receptors effecting dopamine regulation
	Eliprodil		
	Fluoxamine	Clinical	Reduction of σ_1_R receptors in the postmortem schizophrenic brain Adjunctive medication of σ_1_R ligands effective for cognitive deficits of schizophrenia
Major depressive disorder	Fluoxamine SA4503	Nonclinical	σ_1_R ligands show antidepressive effects in the forced swimming test Neurosteroids, considered as endogenous σ_1_R ligands, show antidepressive effects
	Igmesine		
	Neurosteroids		
		Clinical	Psychotic major depression is improved by fluvoxamine monotherapy
Obsessive-compulsive	Fluoxamine	Nonclinical	Fluvoxamine improves marble-burying behavior in mice through σ_1_R activity
disorder		Clinical	Fluvoxamine effective for obsessive-compulsive disorder
			Fluvoxamine enhances the effect of cognitive behavioral therapy
Alzheimers disease	Donepezil	Nonclinical	Donepezil show neuroprotective properties against Aβ_25-35_ peptide-induced toxicity
			Donepezil show anti-amnesic effects which are antagonized by σ_1_R antagonists
		Clinical	Decrease of σ_1_R receptors in the Alzheimers disease brain

#### Memory loss

At the behavioral level, σ_1_R agonists are antiamnesic (602,776–779) and improve the cognitive abilities in experimental animals via the cholinergic system (777,780). In studies using amnesic rodents, the animals’ amnesia seemed to be alleviated by σ_1_R agonist ligands (781). Examples include PCP-induced cognitive dysfunctions, and amnesias induced by scopolamine (782), the Ca^2+^ channel blocker nimodipine or carbon monoxide (212). In addition, σ_1_R agonists show an enhanced efficacy in animal models of AD-related learning impairments or DEP responses (783,784).

The cognition-improving action of neurosteroids has been shown to be mediated via σ_1_R (181,212). Indeed, σ_1_R ligands and related neurosteroids interfere with the cocaine-induced state of memory loss (785) mediated through inhibition of iNOS (365). σ_1_R agonists also have a similar effect (376).

#### Neuroprotection

**Figure 9.**
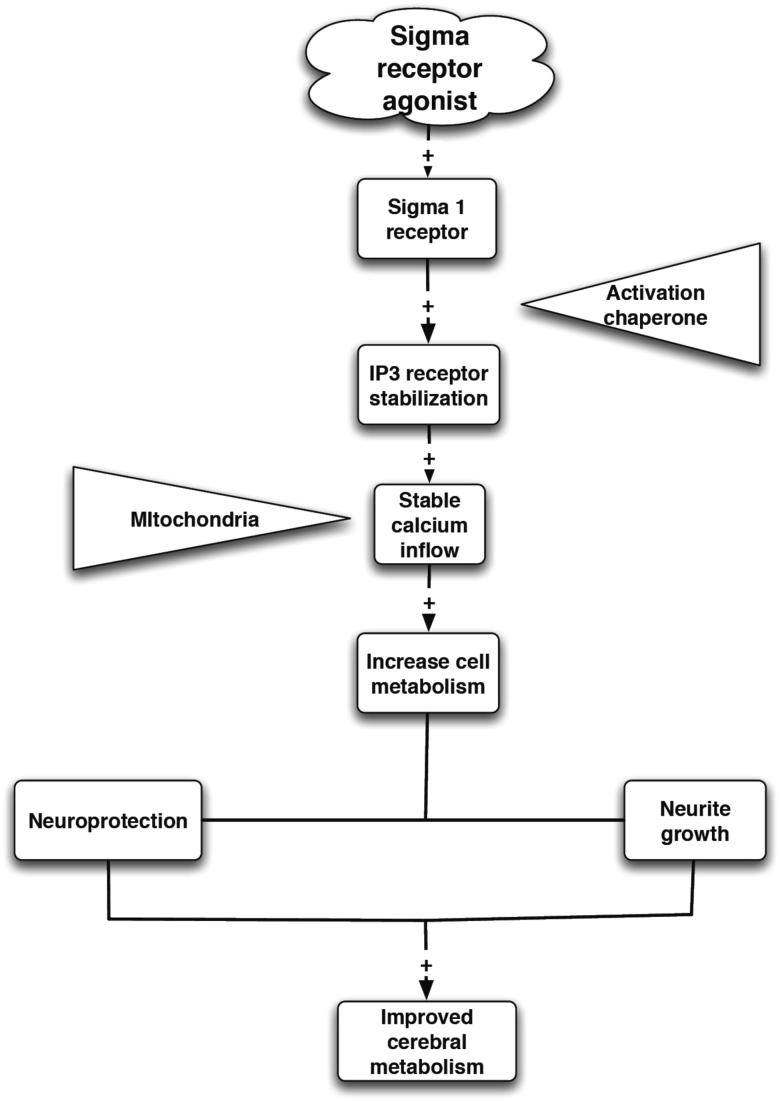
The basic mechanism of neuroprotection by σ_1_R agonists.

The detailed mechanism by which σRs protect the nervous system is not clear (786). The basic mechanism of neuroprotection can be seen in (Figure 9). At least two subtypes of σ_1_R may affect differentially the Glu-medicated NMDA neurotransmission in the terminal and origin regions of the mesolimbic and nigrostriatal DA-ergic systems. There also probably exists a functional interaction between σ_2_R and NMDARs in the hippocampus (360). However, there is some question as to whether the interaction is direct or indirect. Even so, administration of a σ_1_R agonist delays middle cerebral artery occlusion induced neurodegeneration and white matter injury (787), thus confirming the neuroprotective effect of the σ_1_R. Similarly, σR agonists have been show to attenuate brain injury after experimental focal cerebral ischemia in several species (788). The current hypothesis is that σ_1_R agonists protect neurons by a mechanism involving the anti-apoptotic protein bcl-2 (27).

#### σRs and neurogenesis

Recent evidence has shown hippocampal atrophy can persist long after CNS damage is resolved resulting in major depression (789–792). This atrophy could be due to a regression of dendritic processes, an inhibition of neurogeneration or the loss of hippocampal neurons (793). It has also been shown that hippocampal atrophy can be reversed by successful antidepressant treatments and that *in vitro*, classical antidepressants promote neurogenesis (794). σ_1_R has a role in cell morphological changes, specifically in the initiation of neurite outgrowth and sprouting (164,185). In fact, in addition to noted neuronal regeneration, functional recovery has been described following SA-4503 administration (32). Additional support for the neuroprotective effects of σ_1_R is the finding that mutations of the receptor are associated with frontotemporal lobe degeneration and MND (41).

σ_1_R and ankyrins are highly concentrated in the growth cone of NG-108 cells, a region related to neurite sprouting, extension and guidance (164). The σ_1_R agonist (+)- PTZ has no effect by itself on neurite sprouting, but potentiates the neurite-sprouting, induced by nerve growth factor [NGF] (185). In contrast, neurite sprouting, induced by cAMP in PC12 cells, is not affected by (+)-PTZ. The σ_1_R antagonist NE-100, regardless of the presence of NGF, does not affect neurite sprouting, but antagonizes the potentiation induced by (+)-PTZ, thus clearly indicating mediation via σ_1_R (185).

Interestingly, similar to σR agonists, the antidepressants imipramine and fluvoxamine potentiate the effects of NGF induce neurite sprouting in PC12 cells (185). These effects of imipramine and fluvoxamine were antagonized by NE-100, while no concentration of 5-HT tested affected neurite sprouting induced by NGF (185), suggesting that the effect on NGF-induced neurite outgrowth of both σ_1_R agonists and classical antidepressants are mediated by σ_1_R. Moreover, cell treatments with NGF, even in the absence of σ_1_R agonists, increased the level of σ_1_Rs in a dose-dependent manner, and the effects of (+)-PTZ and NGF were additive (185).

Interestingly, treatment with imipramine and fluvoxamine also increased σ_1_R. In another *in vitro* model, MT40 cells expressing high levels of σ_1_R, NGF was found to be more potent in inducing neurite sprouting, whereas treatments with σ_1_R antisense DNA significantly reduced the degree of neurite sprouting (176,177,185). Together, these data suggest a primary role for σ_1_R ligands in enhancing NGF-induced neurite growth.

One member of the neurotrophin family, brain derived neurotrophic factor [BDNF], has been heavily implicated in the actions of antidepressants, and perhaps σR agonists, since chronic treatments with a variety of antidepressant therapies induce an increase in BDNF expression (795–798). Moreover, BDNF administration itself has been shown to produce antidepressant effects in behavioral models of depression (799–801).

The effects of σ_1_R ligands on BDNF expression need to be defined further. Thus far, this has only been studied with the σR ligand E-5842, which showed no effects on BDNF or NGF levels following chronic treatments (802). However, E-5842 presents an σR antagonist profile, so its lack of efficacy cannot be considered as an indication of what might be the effects generated by σ_1_R agonists. It is still conceivable that σ_1_R agonists would potentiate the effects of BDNF, similarly to that observed previously with NGF.

Another growth factor of importance is epithelial growth factor [EGF] (803). EGF is present in the CNS and known to stimulate cell proliferation in PC12 cells. A recent report indicated that in PC12 cells, the overexpression of σ_1_R induces a three-fold increase in neurite sprouting. This effect is suppressed by the σ_1_R antagonist NE-100 (176,177). The overexpression of the σ_1_R in squamous cell carcinomas has shown a strong positive correlation with tumor node metastases [TNM], indicating a potential prognostic tool based on pathology assessment and σ_1_R expression (804). In the context of this review, these data are even more interesting if one considers that EGF has been shown to enhance NMDA-induced modulation of intracellular Ca^2+^.

More research will be required to elucidate the exact basis for the observed potentiation of neurotrophic effects by σR agonists and whether σR ligands always affect neuronal survival and neurogenesis (805).

#### Depression, antidepressants and stress

There are a number of possible mechanisms of action for σR ligands to act as antidepressants, including σR, Glu, 5-HT neurotransmission and Ca^2+^ regulation (26,806). σ_1_R ligands may present a novel mechanism of antidepressant action with potential for a faster onset of action than classical antidepressants (805,807) and SSRI drugs (55,808).

Depression often coexists with cardiovascular diseases, such as hypertension and heart failure, in which sympathetic hyperactivation is critically involved. Reduced σ_1_R brain function in depression decreases heart rate via neuronal activity modulation (809). Reduced brain σ_1_R exacerbates heart failure, especially when combined with pressure overload via sympathetic hyperactivation and worsening depression (810).

The first interest in σR ligands as antidepressants originated from the observation that the antidepressants fluvoxamine (811,812), fluoxetine (813), citalopram, sertraline, clorgyline and imipramine all possess moderate to high affinity (Ki 36–343 nM) for σ_1_R sites (655,659,660,814). Antidepressant treatments, or other modifications of the 5-HT system, induce changes in σR binding properties. For example, repeated treatments with the TCA imipramine (14 days) causes a decrease in the total number of σ_1_R binding sites without affecting the affinity of [^3^H]DTG binding to σR sites in the striatum, hippocampus and cortex of the rat (800,815). Therefore, certain differences in the clinical effects of various antidepressants may, in part, be explained by their distinct influence on cerebral σRs (800,815).

More direct evidence of the potential antidepressant properties of σR ligands was obtained from behavioral experiments. SA 4503 (359), (+)- PTZ, DTG, JO-1784 and SKF-10,047 agonists decrease in a dose dependent fashion the immobility in the FST, whereas the σR antagonists NE-100 and BD1047 blocked these effects (181,208,816). In addition, SA 4503 and (+)-PTZ also decreased immobility time in the Tail Suspension Test [TST], an effect also antagonized by NE-100 (817). Interestingly the antidepressant-like effect of SA 4503 in the FST, a test of a rodents behavioral response to the threat of drowning, was potentiated by the non-competitive NMDA antagonist amantadine (129).

OPC-14523, a combined σ_1_R and 5-HT_1A_ receptor ligand (323), decreases immobility time where the effect of OPC-14523 can be enhanced by its daily administration for 7 days using the FST as a behavioral biomarker (324). Both the σ_1_R antagonist NE-100 and the selective 5-HT_1A_ antagonist, WAY 100635 (818) antagonized the behavioral effects of a single dose of OPC-14523 in the FST (324).

Moreover, a one-week pretreatment with *para*(4)-chloroamphetamine [p-CPA] depletion of brain 5-HT, failed to diminish the antidepressant effects of OPC-14523 in the FST (819), suggesting that σRs alone can mediate the antidepressant effects produced by OPC-14523 and that the combination of the σR and 5-HT_1A_-receptor activity could induce a more potent or rapid “antidepressant-like” effect.

In keeping with this hypothesis, a potentiation of the “antidepressant-like” effects in the rodent FST has been observed following the combined administration of σR and 5-HT_1A_-receptor agonists compared with their separate administration (820). In the chronic mild stress behavioral [CMS] model (chronic stress is believed to be involved in the etiology of affective psychiatric disorders), the σR ligands SKF-10,047 (821) and DEX reversed the motor suppression induced by stress (767,805).

Most of the data regarding σR and depression have focused on the σ_1_R; however, the σ_2_R ligand Lu 28-179 also has shown “antidepressant like” activity in the CMS model of depression. Specifically, three-week treatments with antidepressants led to a normalized sucrose intake in rats, which reversed the decreased intake caused by the stress. Lu 28-179 did not affect sucrose intake in non-stressed controls, but produced a significant increase in sucrose intake in rats exposed to CMS (822,823). However, even if Lu 28-179 has a higher affinity for σ_2_R, it also has affinity for σ_1_R (822); therefore, a role of the σ_1_R in these “antidepressant-like” effects of Lu 28-179 cannot be excluded.

In animal models, neurosteroids with affinity for σRs have also been shown to exert “antidepressant-like” effects that are dependent on the endogenous neurosteroidal systems. For example, the effect of JO-1784 σR agonist on the FST was enhanced in ADX/CX mice compared to control animals, whereas another σR agonist, PRE-084 (127), demonstrated a significant antidepressant effect only in ADX/CX mice (181); however, this effect has been reported more recently in C57BL/6J and to a lesser degree in Albino Swiss mice (824).

The σ_1_R-antagonist BD 10047 (208) blocked all these effects (181). Furthermore, treatments with finasteride, which lead to the accumulation of PROG, also blocked σ_1_R-mediated antidepressant effects. Thus, as discussed previously, circulating steroids appear to exert a tonic modulatory effect on the σ_1_R and therefore on σ_1_R-mediated “antidepressant-like” effects (181). It follows that the potency of σ_1_R agonists as antidepressants is highly dependent on the endogenous PROG levels. Depressed patients such as the elderly with decreased levels of neurosteroids, which would be tonically inhibiting σR to a lesser degree, might be particularly sensitive to such treatments (181).

Only a few controlled clinical trial data are available regarding the effect of σR ligands in depressed patients (825). The results from a double-blind placebo controlled study, obtained from an interim analysis, showed that a dose of 20 mg/day of JO-1784 was superior to placebo and to 20 mg/day of fluoxetine. However, at 100 mg/day, JO-1784 was not different from the placebo (206), which is in keeping with the dose response curves mentioned above (206). A phase II study of SA4503 (cutamesine) in patients with major depression is currently underway (121).

Therefore, even if very limited, the clinical data support the hypothesis that σR agonists could be effective antidepressant medications. However, the mechanisms of action through which σR ligands could exert their antidepressant effects have not been clearly identified. Recent work points to the involvement of the executive function of the PFC (826). As attention deficit disorder [ADD] responds to the stimulant methylphenidate operating via the σ_1_R (827), investigation of these stimulant effects might help elucidate the mechanism of action of σR ligands in depression.

Attempts to identify the mechanisms by which σR ligands exert their effects have brought to light the role of σRs in the regulation of Ca^2+^ (153,161,164,165,376) or K^+^ signaling (88,133,138,139). The effects of JO-1784 in the FST were demonstrated to be Ca^2+^-dependent, since the extracellular Ca^2+^ chelator ethylenediamine tetraacetic acid [EDTA] prevented the effect of JO-1784 in a dose-dependent manner. In addition, a lower dose of JO-1784 had no effect by itself, but co-administered with the l-type voltage-dependent Ca^2+^ channel [VDCC] positive modulator (−)-Bay K8644, it significantly reduced immobility time in the TST.

In agreement with the hypothesis that σRs affect Ca^2+^ regulation, the l-type VDCC antagonist, verapamil and the *N*-type VDCC antagonist, α-conotoxin, blocks the effects of JO-1784 (181,249–251). Therefore, σ_1_R may be interacting with pre- or postsynaptic VDCCs to exert antidepressant-like effects in the FST (181).

Bradykinin, which increases IP_3_ levels, enhances the effect of JO-1784 (249), whereas the IP_3_R antagonist, xestospongin C, blocks the effect of JO-1784. Thus the mobilization of intracellular Ca^2+^ from IP_3_R-sensitive pools appears to participate initially in the behavioral effects mediated by σ_1_Rs located on the ER membranes (249). The σ_1_R then putatively moves to the plasma membrane and interacts with the VDCCs (154,161).

It is likely that σR ligands’ ability to modulate both Glu and 5-HT transmissions also contribute to the antidepressant-like effect observed in behavioral models. The molecular mechanism underlying σRs’ ability to modulate 5-HT and Glu-ergic transmissions may involve σRs’ ability to modulate Ca^2+^. This modulation could represent a secondary target involved in the effects of σR ligands on both the Glu and the 5-HT systems, thus leading to one primary target (377,828). Recently, practical treatment efforts have shown that σ1Rs are also one of the major pharmacological therapeutic targets of selective serotonin reuptake inhibitors [SSRIs] (829).

#### Schizophrenia and psychosis

Schizophrenia is one of the most devastating diseases for both the affected patient and those close to him or her. In the search for medications the antipsychotic effect of stimulation σ_1_Rs has been investigated (830). Experimentation with a number of typical and atypical antipsychotics has been investigated, but the SIGMAR1 gene (σ_1_R gene) does not confer susceptibility to schizophrenia (831). Interestingly, σ_1_R polymorphism is associated with an increased risk of schizophrenia and differential activation of the PFC (344) and the severity of AD (44).

The effect of chronic administration of the atypical antipsychotic E-5842, a preferential σ_1_R ligand, on iGluR subunit levels of mRNA and protein reveals differentially regulated levels of the NMDA_2A_ and of GluR_2_ subunits in a regionally specific manner. Concentrations of immunoreactivity for the NMDA_2A_ subunit are unregulated in the medial PFC, the frontoparietal cortex, the cingulate cortex and in the dorsal striatum, while they are down regulated in the nucleus accumbens. Concentrations of the GluR_2_ subunit of the AMPAR are up regulated in the medial PFC and the *nucleus accumbens* and down-regulation is observed in the dorso-lateral striatum, indicating that E-5842 is able to modify levels of several GluR subunits (15,380,802).

#### Psychosis

Psychosis occurs in 10% to 37.1% of patients with mood disorders (832,833). Psychotic depression is a clinical subtype of major depressive disorder and is characterized by psychosis accompanied by greater severity of depressive symptoms that include psychomotor impairment (retardation or agitation), morbid cognition (involving guilt and a sense of deserving punishment), suicidal ideation and neuropsychological impairment (834,835). Psychotic depression has been shown to have poor prognosis when compared to nonpsychotic depression (i.e. higher rates of recurrence, greater incapacitation, more frequent hospitalization, longer episodes and greater mortality) (836–839). Although several reports suggest abnormalities of endocrine, DA-ergic and serotonergic systems in psychotic depression (840,841), pathophysiology of psychotic depression is still unclear.

Psychotic depression has traditionally been treated with electroconvulsive therapy and classical antipsychotics, such as respiridone (842), in conjunction with tricyclic antidepressants, such as desipramine (843), although tardive dyskinesia may occur following protracted exposure (844). More recent studies have demonstrated the efficacy of atypical antipsychotics and SSRIs in treating psychotic depression (845,846).

Interestingly, SSRI monotherapy, especially fluvoxamine (Luvox), has been shown effective against both the psychotic and depressive symptoms of this disorder (845,847–851). Based on these findings, it has been recently proposed that SSRIs might have multiple action sites in the brain, in addition to serotonin transporters: perhaps σ_1_Rs might play a role in the therapeutic action of SSRIs (752).

Some studies have demonstrated the possible link of psychotic depression to dysregulation of neurotransmitters, such as DA and 5HT; abnormality of brain lipid ganglioside; or hyperactivation of the neuroendocrine and the DA system (840,852). In addition, some studies have suggested that the abnormality of cortisol responses to the dexamethasone suppression test is more prevalent in psychotic depression than in nonpsychotic depression (837,838,841); hence it is possible that psychotic symptoms in depression could be due to increased DA activity secondary to hypothalamic-pituitary-adrenal [HPA] axis over activity (853,854). The observation that psychotic depression frequently appears in patients with neuroendocrine diseases such as Cushings syndrome supports the involvement of the abnormalities of the endocrine system in psychotic depression (855–858).

As previously stated σ_1_R antagonists show antipsychotic effects *in vivo*. Although some σ_1_R antagonists have been shown to inhibit apomorphine- or amphetamine-induced behavioral alterations (859), other studies clearly show that selective σ_1_R antagonists more specifically inhibit the PCP-induced behaviors (156,771).

σ_1_R antagonists rimcazole and BMY-14802 have been tested in clinical trials of acute psychotic symptoms of schizophrenia, but the antipsychotic actions of these compounds have not been confirmed (860,861). The synthesized σ_1_R ligands SL82.0715 and EMD 57445 (panamesine) have been shown to improve negative symptoms in open clinical trials (862–864).

Fluvoxamine (Luvox), showing the utmost potent effectiveness in the treatment of psychotic depression, has the highest affinity for σ_1_R (*K_i_* = 36 nM) among SSRIs (655). Indeed, the efficacy of SSRIs in psychotic depression appears to correlate better with their affinities for σ_1_R than with those for 5HT transporters (655,849,865). Chronic fluvoxamine exposure *in vitro* causes an up regulation of σ_1_R and potentiates the neuritogenesis in a σ_1_R-dependent, but 5HT-independent, manner (185). Studies have also demonstrated that chronic fluvoxamine increases, in a 5HT-independent manner, ALLO in rat brains and in the cerebrospinal fluid [CSF] of patients with depression (866,867). One study demonstrated a statistically significant correlation between symptomatology improvement and the increase in ALLO following fluoxetine (Prozac) or fluvoxamine treatment (867).

Selective σ_1_R ligands potently stimulate adrenocorticotropic hormone release after central or peripheral administrations in rats (868,869). Therefore, it is possible that one of the action sites of fluvoxamine may involve σ_1_R that regulate the neuroendocrine system in the brain (870).

#### Seizures

Seizures associated with cocaine intoxication are a serious clinical problem requiring immediate and adequate treatment. The seizures appear to arise from the interaction of cocaine with GABAergic and Glu systems (871). Accordingly, pharmacological studies have demonstrated that GABA_A_R agonists and NMDAR antagonists can efficiently inhibit cocaine-induced seizures, whereas Na^+^ and Ca^2+^ channel blockers were ineffective (457). The likely interactions are extremely complex; hence, looking at one component in isolation could be misleading.

An involvement of 5-HT_2_, DA and σRs in cocaine-induced seizures has also been proposed (872). Some of these changes, such as expression of immediate early genes and increase in neuropeptide biosynthesis may play a compensatory anticonvulsive role; however, other alterations e.g. up-regulation of NMDARs may increase susceptibility to seizures (538). Stimulation of σRs down-regulate electro-acupuncture induced seizures (873). In fact, sigma receptor-mediated events may play some role in seizure processes in the central nervous system and can modulate the protective activity of some conventional antiepileptic drugs (874).

#### Pain

Although no specific σR ligand has reached the market for the treatment of pain, different pharmacological approaches to the alleviation and treatment of pain have been investigated using σ_1_R agonists and antagonists (15,38,875–879), particularly regarding potential interaction with opioid analgesics and the effect on analgesia (302,880). Activation of σ_1_Rs antagonizes opioid analgesia (881,882), where antagonists potentiate opioid analgesia (681,883). σ_1_Rs differentially modulate acute vs. chronic pain (884–887) and possibly migraine headaches (888): they are also involved in visceral pain (889). In fact, the σRs have been proposed as a modulatory system influencing the analgesic activity of opioid drugs (39). The most promising effects of the σRs lie in the potentiation or modification of the action of other analgesics such as acetaminophen (890) and morphine (891). Such observations may provide a starting point for the development of novel analgesics.

Work has been carried out to identify the mechanism of action of σRs in pain (892). The findings that EAAs have actions on σRs indicated that the EAAs might act via the Glu system in the transmission of nociceptive information (483). In fact, NMDARs receptors play an important role in the potentiation of morphine antinociception (893), and it has been shown that activation of spinal σ_1_R enhances NMDA-induced pain via PKC- and PKA-dependent phosphorylation of the NMDA receptor NR_1_ subunit (458,894).

#### Addiction

Many drugs of abuse, including cocaine and METH, produce effects that can be mitigated through σRs, particularly the σ_1_R subtype (872) including neurotoxicity (12); hence, it has been suggested that σ_1_Rs should be considered as potential compound for substance abuse (895). More specifically, agonists at σ_1_R and σ_2_R inhibit NMDA-stimulated DA release from motor and limbic areas of rat brain (896). Both cocaine and METH exhibit significant affinities for σRs, and about a 10- to 20-fold preference for the σ_1_R subtype (897,898). Because of these effects, it has been suggested that σR antagonists are an obvious potential medications for the treatment of drug abuse (872), and CM156 has been shown to attenuate the neurotoxic effects of METH (13).

These interactions appear physiologically relevant because treatment of animals with selective σR antagonists significantly attenuates cocaine-induced locomotor activity, conditioned place preference, behavioral sensitization, convulsions, lethality and changes in gene expression (897,899–902). The importance of the σ_1_R subtype is supported by the ability of antisense oligonucleotides against them to prevent a number of cocaine-induced behaviors including locomotor hyperactivity, conditioned place preference and convulsions (897,902).

Under normal conditions the brain maintains a delicate balance between inputs of reward seeking controlled by neurons having the D_1_-like family of dopamine receptors and inputs of aversion coming from neurons having the D_2_-like ones (663). Cocaine is able to subvert these balanced inputs by altering the cell signaling of these two pathways such that D_1_ reward seeking pathway dominates. D_2_ receptors (the long isoforms of the D_2_ receptor) can complex with σ_1_Rs, a result that is specific to D_2_ receptors; thus, signaling via D_2_ receptor containing neurons, destabilizes the delicate signaling balance influencing drug seeking that emanates from the D_1_ and D_2_ receptor containing neurons in the brain (663).

Antagonism of σRs, using either putative antagonists or antisense oligonucleotides, also reduces METH-induced locomotor activity and behavioral sensitization (754,898). In addition, σR proteins levels become up regulated in the brains of rodents who self-administer or are repeatedly injected with METH (71,903). Despite the known interactions between σRs and psycho stimulants such as cocaine and METH, other than an early abstract reporting the binding of 3,4-methylenedioxymethamphetamine [MDMA] to σRs, no other studies to investigate this interaction have been conducted (14). METH and MDMA are structurally similar so the question of whether the interaction between σR and MDMA is similar. Experimental evidence has now shown that indeed the interaction is similar (14).

σ_1_Rs are critically involved in the rewarding effect of cocaine (900,904). Cocaines mechanism of action involves initial inhibition of neuronal monoamine transporters primarily in the DA reuptake systems located on mesolimbic neurons. Cocaine rapidly increases the DA neurotransmission and triggers adaptive changes in numerous neuronal circuits underlying reinforcement, reward, sensitization and the high addictive potential of cocaine (122,784).

There appears to be regional differences as to the up-regulation of the σ_1_R. At present the major up-regulation has been recorded in the regions involved in addiction and reward (899). The observation that repeated administration of cocaine rapidly provokes over expression of the σ_1_R outlines its major role in these first psychological steps of addictive processes (905). Indeed, there is little question that the behavioral effects of cocaine can be related to the σ_1_R (897).

*In utero* cocaine [IUC] exposure results in offspring rats with complex neurochemical and behavioral alterations, particularly affecting learning and memory processes (871). However an investigation into the impact of IUC exposure on memory functions in male and female offspring rats revealed that the activation of the σ_1_R neuromodulatory receptor *in utero* allows a complete behavioral recovery of the memory functions in prenatally cocaine-exposed rats (779).

#### Neurodegeneration

Seizure activity, by overstimulation of the σR is mediated via iGlu, particularly NMDAR; this has been associated with neurodegenerative processes such as status epilepticus (906,907), cerebral ischemia (908), perinatal asphyxia and traumatic brain injury (407).

Because the iGluRs are ion-gated channels selective to Na^+^, K^+^ and Ca^2+^, any sustained stimulation of the GluRs results in osmotic damage due to the entry of excessive ions, in particular Ca^2+^ and water. The entry of this material results in apoptosis and necrosis in neurons (382,384–386,394,412,438,511).

This increase in the intracellular Ca^2+^ concentration in neurons is crucial to the determinant of injury that occurs following activation of several enzyme pathways and signaling cascades including as phospholipases, PKC, proteases, protein phosphatases, nitric acid synthases, oxidative stress (423) and the generation of oxygen-based free radicals [ROS] (382,384,386,387,394,412,415,428,486,489,490,491,543,909,910,911). Activation of the σ_1_R inhibits glutamate-induced death of neuron by reducing ROS (912,913).

Neurons are not the only cell type in the nervous system to be damaged by high concentrations of Glu (914). Functional NMDAR recently have been reported in brain glia (915), astrocytes (247,916) and oligodendroglia (409,559,917,). Glial and neuronal NMDARs are functionally and structurally different from the neuronal NMDR; however, the structure of σRs in these cell types is not known at present, and it can only be speculated that the alteration of σRs in these cell types would ameliorate damage caused by overstimulation of NMDARs.

Activation upon ischemia triggers Ca^2+^-dependent damage of oligodendrocytes and myelin (559,918), a finding that has implications for many central nervous disorders such as MS. Because of the association between σR and Glu, it is likely that σRs are involved in exogenous and endogenous Glu toxicity in oligodendrocytes.

### Heart and vessels

σRs have been implicated in the regulation of the cardiovascular system [CV], and σ_1_R transcripts have been found in parasympathetic intracardiac neurons (756) and the human ether-a-go-go-related gene [hERG] channel (919). These structures are central to cardiac excitation and rhythmic control (920–922).

Both σ_1_R and σ_2_R interact with the human ether-à-gogo-related gene (hERG). hERG encodes a cardiac channel that is also abnormally expressed in many primary human cancers, potentiating tumor progression through the modulation of extracellular matrix adhesive interactions. σ_1_R potentiates hERG current by stimulating channel subunit biosynthesis and σ_1_R silencing does not modify hERG mRNA contents but reduces hERG mature form densities. A physical association has been shown in HEK cells expressing hERG and σ_1_R: both proteins co-immunoprecipitate. σ_1_R expression enhances both channel protein maturation and stability (919).

In rabbits, all σ_2_R agonists have been shown to reduce phenylephrine-induced cardiac arrhythmias. They prolonged action potential duration in rabbit Purkinje fibers and reduced human ether-a-go-go-related gene (HERG) K(+) currents. It has been suggested that σ_2_R-receptor ligands block I(Kr) and that this effect could explain part of the antiarrhythmic properties of this ligands family. Nevertheless, an interaction with HERG channels not involving σ_2_R seems to share this pharmacological property. The repolarization prolongation and the early-after depolarization can be responsible for “torsades de pointe” and sudden cardiac death. It is for this reason that particular caution has to be taken using ligands with affinity for σ_2_R with respect to abnormal cardiac function (923,924).

The relationship between depression and heart failure is known, but the mechanism has not been fully elucidated. Depression is associated with a substantial increase in the risk of developing heart failure and is independently associated with increased cardiovascular morbidity and mortality. Reduced σ_1_Rs density in depression decreases heart rate via the sympathetic stimulation in the autonomic nervous system [ANS] (809) and exacerbates heart failure, especially when combined with pressure overload and worsening depression (810). Conversely, cardiovascular disease can lead to severe depression. Thus, therapy with SSRIs used for treatment of depression, has been recommended to reduce cardiovascular disease morbidity and mortality (829,925).

Similarly, GluR have been found in cardiac intramural nerve fibers and ganglia cells as the main structures expressing GluRs in the conducting system (926) and similar findings have been seen in human hearts (500).

These effects of the σ_1_Rs are probably mediated via PKC- and PKA-dependent phosphorylation of the NMDA receptor (458); however, the exact cellular function of σ_1_R in these cells remains to be determined. Regardless, a reduction of brain σ_1_Rs also contribute to sympathetic hyperactivation of the heart (810,927), probably via altered Na^+^ channels (150,928,929). In the reverse, stimulation of brain σ_1_Rs ameliorates hypertrophy in mice (925) and cardiac function following myocardial infarction (927).

σR ligands have been shown to modulate contractility, Ca^2+^ influx and cardiac rate *in vitro* (930,931), where σR stimulation causes changes in beating frequencies, followed by irregular contractions. In this case, changes in Ca^2+^ are not mediated by sarcoplasmic reticulum Ca^2+^ transport systems (923,930).

Experimentally, pre-treatment with an σR agonist improves the reperfusion recovery of cardiac pump function in rat hearts (932) and is cardioprotective (925,933). Activation of the cardiac σR prompts an augmentation of tolerance to the reperfusion damage; however, this effect decreases with time, indicating a possible desensitization of the receptor (934).

In any case, σR activation prevents reperfusion contracture, increases pressure in the left ventricle, and improves survival of cardiac myocytes after ischemia and reperfusion. Conversely, pre-treatment with an σR antagonist augments the reperfusion systolic dysfunction of the myocardium and prevents post-ischemic contractures and cardiac cell lesions (932). Interestingly, the electrical stability in the rat model of post-infarction cardiac sclerosis and stress, activation of either µ- or κ_1_-opioid receptors or blockade of σ_1_R reverses the decrease in ventricular fibrillation threshold (935) increasing the probability of sudden death.

By contrast, l-Glu increases the frequency of Ca^2+^ oscillations in cardiac excitation and rhythmic control (436), which has been positively correlated with increased contraction frequency in myocardial cells. Such an increase may reduce cardiac filling, hypoxia and angina-like chest pains (936,937). It would appear that in the case of the heart, GluR and σRs might have opposing actions.

Activation of σR reversibly blocks the delay in outwardly rectifying K^+^ channels, large conductance Ca^2+^ sensitive K^+^ channels and the M-current. This blockade is dose-dependent suggesting the effect is mediated by σ_1_R activation (930,931). Thus, activation of σ_1_R depresses the excitability of intracardiac neurons and is likely to block parasympathetic input to the heart. σR stimulation has been shown to cause changes in beating frequencies, which are followed by irregular contractions (923), probably mediated through NMDARs. It also has been suggested that in the heart the signal transduction pathway does not involve a diffusible cytosolic second messenger or a G protein (158,756), a finding that is supported (207) and refuted by others (174). Therefore, the activation of σ_1_R is most likely mediated via iGluRs rather than mGluRs.

In addition to myocardial contraction modulation, σRs also are involved in the regulation of coronary and peripheral arterial vascular tension (923). Experiments have shown that the changes in Ca^2+^ induced by σ_1_R stimulation are not mediated by sarcoplasmic reticulum Ca^2+^ transport systems and do not affect the apparent sensitivity of the myofilaments to Ca^2+^ (930). In fact, σR agonists increase the intracellular Ca^2+^ levels by stimulating IP3 production and, thus, modulate contractility (167).

### Muscle and bones

Many neuroleptic drugs reported to play a role in the control of movement bind with high affinity to σ_2_R. The high affinity of some neuroleptics for these sites suggests their possible involvement in some σ_2_R-mediated side effects, such as drug-induced dystonia (938). A correlation between the clinical incidence of neuroleptic-induced acute dystonia and binding affinity of drugs at the σR, indicate that the σR might be involved in neuroleptic-induced acute dystonia, which has been confirmed by σR agonist induced neck dystonia of rats (764).

As bone has been shown to express many of the molecules associated with Glu- mediated signaling (939–941), it is probably that σRs are involved in normal bone function as well as in disease states, although there is some debate regarding the role of Glu in controlling bone growth (942–944). Nonetheless, all osteoblasts (943,944), osteocytes and osteoclasts express one or more of the GluR subunits, including NMDARs (492,497,945–948). The Glu Asp transporter [GLAST] has also been identified in bone (492,949,950). As activation of σRs is an integral part of Glu system, it is likely that they act in conjunction with GluRs to affect cellular changes.

### Lung

Considerable data are available for the presence of σRs in lung tissue and a role for them with respect to cancer and chemotherapy (951). In fact, σRs are expressed in a wide variety of tumour cell lines (755,952,953), including non-small-cell lung carcinoma, large-cell-carcinoma (NCI-H1299 and NCI-H838), lung cancer cell line (NCI-H727) (755,953,954) and small-cell lung cancer (NCI-H209/N417) (955). More recently, in material obtained from patients with lung tumors elevated PROG receptor membrane component was seen associated with increased σ_2_Rs levels in the tumor mass and blood plasma (956).

The anatomical sites of σRs in normal lung are likely to be associated with pulmonary nerves (951). However, expression of the σR has been used to visualize cancerous cells in the lung (86,108,676).

The presence σRs in the airway structures such as the larynx, esophagus and mast cells also implicate the GluRs (and probably the σRs) in the mediation of asthmatic episodes (516,957,958). Thus the excitation of GluRs in the air passages may be important in airway inflammation (959) and hyper reactivity observed in bronchial asthma (440,960). Their presence also could explain the enhancement of acute asthmatic attacks by Glu-containing foods (957).

Current antitussive medications have limited efficacy and often contain the opiate-like agent DEX, which is an σR agonist, or antagonist, depending on the dose administered (194,733). The mechanism whereby DEX inhibits cough is ill defined; however, DEX displays affinity at both NMDARs and σRs, suggesting that the antitussive activity may involve central or peripheral activity at either of these receptors. Experimental findings in guinea pigs support the argument that antitussive effects of DEX may be mediated via σR, since both systemic and aerosol administration of σ_1_R agonists experimentally inhibit citric-acid-induced cough (961).

### Endocrine system

Visualization using autoradiography with σR radioligands has revealed these receptors in the rat pituitary, adrenal, testis and ovary (676). The highest density of σRs is present in the ovary, with progressively lower densities present in the testis, pituitary, adrenal and cerebellum, respectively (676). This distribution is not surprising given that studies have found that PROG and DHEAS bind to σ_1_Rs (183,228–230).

σRs are believed to be responsible for important regulatory functions in the endocrine system (222,962,963). However, the role of σRs in endocrine cells remains unclear, particularly given the plethora of possible neurotransmitter interactions in the HPA (964). It has been suggested that endogenous σR ligand(s) would contribute, together with other endocrine factors such as DA, neuropeptide Y, or GABA, to the control of pituitary functions (868).

Because steroids have been shown to interact with σ_1_R (123,210,222) and because they exhibit a significant physiological relevance in the modulation of the electrical activity of frog melanotrope cells (965,966), it can be hypothesized that they represent a very interesting class of endogenous σR modulators in endocrine cells.

Because of the many effects of the endocrine system are related to homeostasis, it is not surprising to note that manipulation of the σRs has numerous potential effects (967). For example, long-term administration of neuroleptic agents, such as haloperidol, has been associated with the development of a drug induced syndrome of inappropriate antidiuretic hormone release, which occurs in the absence of other abnormalities in endocrine function (968). Furthermore, it has now been hypothesized that interaction with some neuroleptic agents and the posterior pituitary σR ligands can inhibit K^+^-channel function (138,139).

There are minimal data concerning role of σRs and the involvement of GluRs in diabetes mellitus and associated dysfunctional islet cells (418,435,486–488,512,523,524,669,969–973), and abnormalities of HPA function (507,974,975). It is likely that σRs also play a role in these related disorders.

### Reproduction

σ_1_Rs are expressed in the placenta (976), in spermatozoa (977) and other parts of the reproductive system. As stated previously, the highest density of σRs is present in the ovary, with lower densities present in the testis (676). In the ovary, σRs are seen in highest density in the maturing follicles, and lower densities in resting follicles. In the testis, they are present in highest concentrations in the ductuli efferentes and ductus epididymis. Lower densities of binding sites are present in the seminiferous tubules, but none in the interstitial tissue (676). This pattern is mirrored by the distribution of GluRs (978). In addition, σRs (977) and GluRs (418) are abundant in spermatozoa and may affect they signaling pathways (977) in conjunction with PROG or prostaglandin E_1_ [PGE_1_].

The developing fetus may be indirectly affected by PROG levels, which have been shown to decrease brain σR function (179). Here PROG acts as an antagonist ligand for the σR during pregnancy. At parturition, Glu output from the fetal liver reduces, leading to a fall in fetal arterial Glu concentrations, which correlate with a marked decrease in PROG output from the pregnant uterus (979), with probable up regulation of σRs (179).

Modulation of ion channels in *Xenopus* oocytes was observed in the presence or absence of σ_1_R ligands, suggesting that the σ_1_R may form a functional complex with the expressed ion channels (88). In fact, these authors went on to show that the σ_2_R forms an immunoprecipitating complex with ion channels both in rat neurohypophysis and when co expressed in *Xenopus* oocytes (88).

### Liver and kidney

Liver contains high densities of σ_1_R and σ_2_R (92,980), and these receptors are specifically localized to lipid rafts in rat liver phospholipid membranes (981,982), particularly mitochondria (113,983).

iGluRs and mGluRs also have been demonstrated in the liver (502,520) and mGluRs are involved in the hydrolysis of IP3 and reduction of viable hepatocytes (984). In fact, it has been suggested that GluR is activated by the Glu present in the portal blood and may contribute to toxic liver damage. At present the relationship of σRs and liver disease is still to be elucidated. As a number of σ_1_R agonists are being developed for cancer treatment, especially those with EGFR activity, and as the liver is endodermal in origin, it is likely that a lot more findings will result from further hepatic cancer research (981).

Similarly, the kidney contains high densities of σ_1_R and σ_2_R as determined by using selective σR probes and photo affinity labeling (92). Interestingly, this work, using kidney tissue *in vitro* shows that the 25 and 21.5 kDa proteins represent σ_1_R and σ_2_Rs, respectively. The role of these σRs in renal disease has yet to be determined.

### Eye

Loss of retinal ganglion cells [RGC] is a hallmark of many ophthalmic diseases including glaucoma, diabetes retinopathy, retinal ischemia due to central artery occlusion, anterior ischemic optic neuropathy and may be significant in optic neuritis, optic nerve trauma and AIDS (985). The expression of σ_1_R mRNA in the mammalian retina is greatest in ganglion cells (986), as determined via mRNA expression, cells of the inner nuclear layer, inner segments of the photoreceptor cells and retinal pigment epithelial cells (987). As Glu toxicity is seen mainly in the ganglion cells, a possibility of neuroprotection by σR ligands against ganglion cell Glu toxicity has been suggested (987). In fact, the σR ligand (+)-PTZ prevents Glu-induced apoptosis in retinal ganglion cells (988).

Expression, subcellular localization and regulation of σR experiments have been undertaken in retinal Mueller cells (989). Mueller cells express σ_1_R and demonstrate robust σ_1_R binding activity, which is inhibited by σ_1_R ligands and is stimulated during oxidative stress (913,990). A similar response is seen for σ_2_Rs (991). Additionally, late-onset inner retinal dysfunction in mice lacking σ_1_R has been reported, confirming the importance of σ_1_R in retinal health (992,993). In adult Mueller cells, σ_1_Rs are bound and stimulated under the conditions of oxidative stress, an effect that is amplified when cells were incubated with NO and reactive oxygen species [ROS] (989).

Exposure of lens cells to σR antagonists has been shown to lead to growth inhibition and pigment granule production (994,995), implying that σRs are important during lens development.

### Gastrointestinal system

σR binding sites have been shown to be present in the myenteric plexus of the guinea pig ileum (747) and are important in the regulation of ileal contractions (314), as are the GluRs (64,390,513,518,996,971,997). As Glu and Asp are both involved in regulating acid secretion in the stomach (998,999), it is likely that σRs are also involved.

DTG and its σR-active congeners inhibit electrically or 5-HT-evoked contractions of the longitudinal muscle and myenteric plexus [LMMP] preparation by a neuronal mechanism (314), and as such σR agonists might be possible novel targets for antisecretory therapy in diarrhea (1000). In fact, the importance of σR manipulation in a number of diseases is highlighted by the recent patent applications (1001), where a method of stimulation of salivary secretion using oral administration of certain σR ligands which may be generally described as N,N-disubstituted phenylalkylamine (US Patent 5387614).

σRs induce emesis in a number of species, probably mediated centrally via the vagus as has been shown for Glu, GluR and GLUTs (1002–1004). It is not surprising to note that emesis and nausea are often associated with the use of σR agonists and antagonists (1005).

Unfortunately, nausea is a difficult endpoint to measure in animal studies; hence, most endpoints used with respect to the gastrointestinal system have been limited to vomiting. Nonetheless, symptoms reported by human subjects include nausea following chemical manipulation of σR *in vivo,* but their quality of life scores improved (1006)*.* Nausea and vomiting is probably a common endpoint for EAA poisoning mediated via the Glu, and most likely, σR systems in such poisonings, such as is seen in DomA toxicity (415,418,434,437,439,442).

σRs stimulate physiological motility and inhibit experimentally induced colonic hypermotility. They stimulate the postprandial colonic motility in dogs by acting selectively on sigma receptors located peripherally and probably by affecting the release of cholecystokinin octapeptide through a central adrenergic mechanism (1007). Other findings indicate that σR ligand igmesine, blocks the corticotropin releasing factor and emotional stress-induced colonic hypermotility also via an interaction with central cholecystokinin octapaptide mechanisms (1008,1009).

### Immune system

Pharmacological studies initially identified high-affinity σRs on human peripheral blood mononuclear cells using DTG and haloperidol (677). Subsequent studies employing the σR selective radioligand, [^3^H] (+)-PTZ (600) then identified a lower affinity-binding site on murine B- and T-enriched lymphocytes (1010), human and rat lymphocytes (1011). Here, high concentrations of PCP (gM) compete for binding to σRs on the lymphocytes (98). σ_2_Rs also inhibit T lymphocyte activation (1012).

Evaluation of the effects of σR and the immune system has helped solidify the understanding of the link between the endocrine, nervous and immune systems, although there is still an enormous amount of work required to sort out this relationship (222). σR ligands have potent immunoregulatory properties, including the induction of I l-10 (1013) and the suppression of IFN-γ and granulocyte colony stimulating factor [GM-CSF] (1014).

In murine studies, treatment with σR ligands prevents both graft versus host reactions and delayed-type hypersensitivity granuloma formation (1014). These studies indicate that σR-dependent signaling plays a role in immune-mediated responses. Cocaine, a σ_1_R ligand, is also known to modulate immune function *in vivo* and *in vitro* (1015,1016). σ_1_R has been shown to regulate early steps of viral RNA replication at the onset of hepatitis C virus infection (1017) and a reovirus nonstructural protein σ_1_R is required for establishment of viremia and systemic dissemination (1018).

Because immunocompetent animal models of tumorigenicity and tumor progression can serve as sensitive indicators of immune dysfunction, it has been found that σR ligands do impact host antitumor immunity, probably through a σR-dependent cytokine modulation (1013). Sigma ligands, especially σ_2_R agonists, can inhibit proliferation and induce apoptosis by a mechanism involving changes in cytosolic Ca^2+^, ceramide and sphingolipid concentrations (1019).

Specific cell types that assist in immunoregulation have been investigated σR activity. Studies initially identified high-affinity σRs on human peripheral blood mononuclear cells using DTG and haloperidol (677). Subsequent studies employing the σR selective radioligand, [^3^H] (+)-PTZ (600) have identified a lower affinity-binding site on murine B- and T-enriched lymphocytes (1010).

Previous work has shown high that concentrations (pM) of PCP suppressed lymphocyte proliferation, mitogen-induced IgG and IgM production, and LPS-induced IL-1 production (1020). Another report indicates pM concentrations of PCP and PCP analogues inhibit IL-2 production by concanavalin A [Con A]-stimulated murine splenocytes (1021). Similarly, DTG, haloperidol, (+)-PTZ and (−)-PTZ have been shown to enhance LPS-stimulated murine splenocyte proliferation while PCP was without effect (1010). Lymphocytes do not possess PCP-selective receptors as determined in radio receptor assays using the PCP-selective ligand, [^3^H]N-[1-(2-thienyl)cyclohexyl]-piperidine (677), but a high concentration of PCP (gM) competes for binding to σRs on splenocytes (98).

Regardless of the literature available, it is possible that current research into the effects of manipulation of σRs is not widely known to the public due to proprietary efforts to develop new treatment regimens using stimulation or antagonism of σRs on the immune system and the cells thereof. Due to the many body systems, cell types and substances involved in immunoregulation, tissues that also contain σRs, the manipulation of σRs holds promise for increasing our understanding of immune mediated diseases, cancer and “difficult” infections in which immune dysregulation is an essential part of the pathogenesis of the disease, e.g. HIV and AIDS.

### Neoplasia

Pharmaceutical agents acting at the σR have been used in the treatment of cancer and are receiving considerable attention (1022). A large number of drugs are known to bind with high affinity to σ_2_Rs and these receptors are overexpressed in many cancer tissues, suggesting potential applications for σR ligands in cancer diagnosis and therapy (1023). The potential and specific signal transduction pathways and mechanisms involved in the actions of σR ligands in cancer biology include modulations of the plasma membrane and lipid raft components, intracellular Ca^2+^ levels, cytoskeletal protein functions and ER stress (1022).

#### Expression of σRs in neoplastic cell lines and tissues

Both σR subtypes, σ_1_R and σ_1_R, are highly expressed in tumor cell lines from various human cancer tissues, including, but not limited to, small- and non-small-cell lung carcinoma (755,953–955), large-cell carcinoma (954), renal carcinoma (952), colon carcinoma (952), sarcoma (952), brain tumors (1024), breast cancer (103,755,953), melanoma (755,953), glioblastoma (755,953), neuroblastoma (755,953) and prostate cancer (755,953).

Comparable findings available from rat cancer cell lines, such as C6 glioma (755), N1E-115 neuroblastoma (94,953) and NG108–15 neuroblastoma X glioma hybrid (755), which generally agree with the human data. Many of these observations are based on the binding of labeled σR ligands that are σ_1_R- or σ_2_R- non-specific. In some cases, σ_1_R sites are masked with DEX so as to determine the relative amounts of σ_1_R and σ_2_R sites in the cell preparations. However, these results await confirmation by Western blotting and reverse transcription PCR [RT-PCR] studies (Table 6).

**Table 6.** σR drug binding in tumor tissues and cell lines.

Cell line or tumor tissue	σR ligands tested	Reference
Non-small-cell lung carcinoma	IPAB, haloperidol, DTG	(954)
Large-cell-carcinoma (NCI-H1299 and NCI-H838)	IPAB, haloperidol, DTG	(954)
Lung cancer cell line (NCI-H727)	IPAB, haloperidol, PTZ, DTG, (+/−) dextrallorphan	(755,953)
Breast ductal carcinoma (T47D)	PTZ, DTG, (+/−) dextrallorphan	(755,953)
Renal carcinoma	DTG	(952)
Colon carcinoma	DTG	(952)
Sarcoma	DTG	(952)
Brain tumor tissue	DTG	(1024)
(Nude mouse) neuroblastoma and glioma	DTG	(1024)
Rat neuroblastoma(NIE-115), rat glioma (c6)	PTZ, DTG, (+/−) dextrallorphan	(755,953)
U-138MG glioblastomas	PTZ, DTG, (+/−) dextrallorphan	(755,953)
Breast cancer cell line (MCF-7; T47D; SKBr3)	Haloperidol, CB-64D, CB-184, IPAB	(103,1025)
Small-cell lung cancer (NCI-H209/N417)	IBP, haloperidol	(955)
Neuroblastoma [BE(2)]; SK-N-SH)	PTZ, DTG, (+/−) dextrallorphan	(755,953,1026)
Prostate tumor cells (DU-145) (LnCap)	IPAB, PTZ, DTG, (+/−) dextrallorphan	(755,953,1025)
Mammary adenocarcinoma (line 66)	DTG	(1027)
Melanoma (A375)	PTZ, DTG, (+/−) dextrallorphan	(955)
C6 glioma cells	^11^C-SA4503	(1028)

A comparative study on mouse mammary adenocarcinoma revealed that proliferative cells possessed 10 times more σ_2_R than did quiescent cells (1027); hence, the development of pharmaceuticals to block these receptors is a field of endeavor. The density of σR_2_ sites have been evaluated after the stimulation of mitosis and progression through the cell cycle in the human mammary tumor cell lines T47D and MCF-7 as well as in the prostate tumor cell line DU-145 (1025). The results suggest that there is a direct correlation between the binding of the σR drug [*N*-[1α(2-piperidinyl)ethyl]-4-[I^125^]iodobenzamide [^125^I-PAB], moderately selective for σ_1_Rs and proliferative status; and an up-regulation of σR binding sites occurred before mitosis.

Using *N*-[2-(1′-piperidinyl)- ethyl]-3-123I-iodo-4 methoxybenzamide, also moderately selective for σ_1_Rs, another study also found that σ_1_Rs and σ_2_Rs were present at high density on human breast tumor biopsies but virtually absent in normal tissues (980). Expression of the σ_1_R, monitored immunocytochemically, has been suggested as a possible marker for predicting the aggressiveness of breast tumors, in particular, where there was a significant correlation between σ_1_R expression and PROG receptor status (1028,1029). The age-related decrease in PROG may be important in the binding of σ_1_R agonists in tumor cells (759).

#### σR ligands as tumor imaging agents

The high densities of σ_1_R and σ_2_R binding sites in tumor cell lines and tissues are indicative of their involvement in the cellular pathophysiology of cancer, and as such could have diagnostic potential in tumor imaging. In fact, previous work has developed probes for imaging σ_2_R both *in vitro* and *in vivo* (1030). Most of what is known about σ_2_R has been obtained using either radiolabeled or fluorescent probes, or biochemical analysis of the effect of σ_2_R selective ligands on cells growing under tissue culture conditions. Now it has been shown that the PGRMC1 protein complex is the putative σ_2_R binding site (31).

Numerous nonclinical studies have evaluated the usefulness of radiolabeled σR ligands (1031,1032), as tumor imaging agents in melanoma (734,1033–1036,), breast cancer (954,980,1030,1032,1037–1039), prostate cancer (954,1038,1040) and non-small-cell lung cancer in mouse tumor models (1035). These observations suggested that σ_2_R ligands could be effective for tumor imaging, including radiotracers (1041), coupled with techniques such as positron emission tomography [PET] (757) or single-photon emission computerized tomography [SPECT] (737,1025,1027,1031,1040) and two-photon confocal microscopy (1042). Recently, development of these tracers (σ_2_R ligands) has allowed differentiation of tumors from inflammation (1043), especially T cell lymphocytes (1012), or mast cells (1044). Most of these σR ligands are nonselective for the σ_1_Rs and σ_2_Rs, but, more recently, σ_2_R-selective agents have shown the most promise in this regard (1037,1039).

#### Physiology and pathophysiology of σRs in neoplasia

##### Effects of σR ligands on cancer cell proliferation and death

Several studies have tested the potential effectiveness of σR ligands on proliferation of tumor cells *in vitro*. The effects of various σR ligands (e.g. haloperidol, DTG, SKF10047, PTZ and Rimcazole) on the *in vitro* growth of human mammary adenocarcinoma, colon carcinomas and melanomas show promise (1045).

Cellular proliferation is inhibited, and cell detachment and rounding subsequent to cell death are observed by light microscopy. Of the σR ligands tested, the σ_1_R- and σ_2_R-nonspecific rimcazole, and reduced haloperidol, which is the main metabolite of haloperidol in humans (657), were the most potent inhibitors of cell proliferation (1045). Similar inhibitory effects of σR ligands [e.g. *N*-[2-(piperidino) ethyl]-2-iodobenzamide [2-IBP], haloperidol and N-(2-piperidinoethyl)4-iodobenzamide [IPAB] were observed on small-cell lung cancer (NCI-H209 and NCI-N417) cells (955). IPAB or 2-IBP also inhibited the *in vivo* xenograft proliferation of NCI-N417 cells (955).

The question of the mechanism(s) underlying the inhibitory effect of σR ligands on tumor cell proliferation is an important one. The morphological effect of treating C6 glioma cells with various σR ligands (generally σ_2_R- and σ_2_R-nonspecific) has been examined (755,953). These compounds cause loss of cellular processes, assumption of spherical shape and cessation of cell division, and the time course and magnitude of these effects are dependent on the concentration of the various σR ligands used. Continued exposure to σR ligands for 3–24 h results in cell death, although the morphological effects are reversible if the drug is removed shortly after rounding (755,953). Reduced haloperidol also potently inhibited proliferation of WIDr colon and MCF-7 breast adenocarcinoma cell lines, where in these cells, the intracellular Ca^2+^ levels were raised, and apoptosis was observed (166), although a direct link between them has not been shown.

The ability of σ_2_R ligands to induce cell death in the human breast tumor cell lines MCF-7, MCF-7/Adr^−^, T47D and SKBr3 also has been demonstrated (103). Both σ_2_R subtype-specific and σ_2_R non-selective σR ligands result in cell death by a mechanism that involves apoptosis. This has been suggested to be a novel p53- and caspase-independent apoptotic pathway (103).

The effects of σR ligands on cell growth and apoptosis are thought to occur via the sphingolipid pathway. Therefore, it is not surprising to note that σ_2_R ligands applied to MCF-7/Adr^−^ and T47D breast tumor cells induce a dose-dependent increase in ceramide and concomitant decreases in sphingomyelin (103).

Progress is being made in the development of potential treatment modalities. Nanoparticles have been extensively used as carriers to deliver molecules into tumors through the enhanced permeation and retention effect, and to regulate the release of a chemical or biological effector in response to environmental stimuli such as temperature or pH change. In these cases, cell uptake of nanoparticles has been studied to maximize their delivery into the target cells (1046). Recently, the surface of gold nanocages was functionalized with SV119, a synthetic small molecule specific to σ_2_R, and then was shown to be effective in for targeting cancer cells (1047).

##### Possible mechanisms of σR signal transduction and relevance to cancer cell biology

Although there is considerable evidence for the involvement of σRs in cancer cell biology, the mechanism(s) through which these effects occur has not fully been discerned. As has been discussed previously, σRs have been implicated in a wide range of functions, and formulating a unifying hypothesis for the molecular physiology of σRs to account for all of the varied functions will be a great challenge. Few reports exist that deal directly with the mode of action of σRs.

The homology between the σ_2_R and the sterol isomerase, *ERG2*, of yeast is interesting, given that both the σ_1_R and the sterol isomerase have high affinity for σ_1_R ligands (1048). However, the σ_2_R has never been demonstrated to possess sterol isomerase activity. On the other hand, emopamil-binding protein, which also binds σR ligands, was found to complement a yeast strain containing a deletion of the *ERG2* gene and is a sterol isomerase like ERG2 (147).

##### Modulation of ion channels

Ion channels are expressed in cell lines derived from several different cancer types and can play an important role in metastasis, an integral aspect of which is the control of cell growth and proliferation (1049–1052). The dual observation that σR expression is increased in tumor cell lines or tissues, and that σ_1_Rs act as secondary subunits for some ion channels including Cl^−^ channels (1053) might be of importance, given the accumulating evidence for the involvement of different types of ion channels in proliferation (1049,1050,1052) and metastatic activities of cancer cells (1050,1054–1056). Because down-regulation of K^+^ channel amplitude has been associated with the metastatic phenotype in human prostate and breast cancer (1049,1057), such an effect could underlie the proposed association between cancer progression and σR ligands.

In addition to roles such as proliferation (1057–1059), there are a number of ways in which ion channel activity may contribute to the cancer cell behavior, including migration (1060), apoptosis (1061), adhesion and cytoskeletal organization (1013,1062,1063) and secretion (1064). It remains to be determined whether ion channels, such as the voltage-gated Na^+^ channel (1050,1064–1067) are also modulated by σR ligands in the cancer process.

##### Modulation of Ankyrin

σ_1_R may play a role in controlling the functioning of cytoskeletal proteins (46,66,164). Using immunocytochemical techniques, σ_1_R, ankyrin B and IP3R have been co-localized in perinuclear areas and areas of cell-to-cell communication. It has been proposed that this trimeric complex may regulate Ca^2+^ signaling (164). Although the exact underlying molecular mechanism has not yet been described, it is well known that adhesion and cytoskeletal organization are important factors in cancer cell biology (1068,1069).

##### Modulation of Intracellular Ca^2+^

Evidence suggest that σR in neuroblastoma cells may use Ca^2+^ signals to produce cellular effects (95). By using σR-inactive (but structurally similar) ligands, σ_2_R-selective agents such as CB-64D, and σ_1_R-selective agents have shown that a fast and transient release of Ca^2+^ from the ER is induced specifically by the action of the σ_1_R and σ_2_Rs (1070). In turn, intracellular Ca^2+^ modulation can affect PKC activity. Indeed, in rat brain synaptosomes, DA transporter activity is modulated by σ_2_R ligands via activation of PKC (354). Because intracellular Ca^2+^ signaling is broadly important for many cellular processes, this may be an important mechanism through which σ_2_R ligands produce their documented effects on cancer cells.

##### Modulation of sphingolipid levels

Sphingolipid levels in MCF-7/Adr^−^ and T47D breast tumor cell lines have been investigated following application of σ_2_R specific agonists in order to understand further the molecular mechanism by which σ_2_R ligands could cause their observed morphological and apoptotic effects in various cancer cell lines (103). CB-184 causes a dose-dependent increase in ceramide levels and concomitant decrease in sphingomyelin within the MCF-7/Adr^−^ and T47D breast tumor cell lines.

These effects can be attenuated by *N*-phenethylpiperidine, a nonspecific σR antagonist. These results suggest that σ_2_Rs may use sphingolipid products to affect Ca^2+^ signaling, cell proliferation and survival (86,103). In fact, imaging of σ_1_R in the human brain using SPECT radioligands has started to investigate whether σRs can be used as prognostic indicators (808), even though it is already known that σ_2_R are potentially useful tumor imaging ligands.

##### Immunological changes

The mechanism by which σRs affect tumor cells has been more recently investigated with respect to immunological alterations (1071). σR agonists in mice promote the *in vivo* growth of a syngeneic lung cancer cell lines, which was accompanied by an increase in IL-10 and a decrease in interferon production in spleen cells and at the tumor site. The tumor-promoting effects produced were abrogated by administration of specific antibodies to IL-10, or by administration of a σ_1_R antagonist, indicating that σ_1_R agonist ligands augment tumor growth via a cytokine-dependent, receptor-mediated mechanism that involves regulation of T helper_1_/T helper_2_ cytokine balance (1071). Most likely, the alteration of immune cells and function will impact the process of carcinogenesis.

#### Vascular effects

As tumors progress to increased malignancy, cells within them develop the ability to invade into surrounding normal tissues and through tissue boundaries to form metastases at sites distinct from the primary tumor. The molecular mechanisms involved in this process are incompletely understood but those associated with cell-cell and cell-matrix adhesion, with the degradation of extracellular matrix, and with the initiation and maintenance of early growth at the new site are generally accepted to be critical (1068). σR ligands have also been shown to inhibit stem cell differentiation (96), and modulate endothelial cell proliferation and can control angiogenesis, which makes them a promising target for oncology applications (239).

#### Apoptosis

Apoptosis is a key process in cancer development and progression. The ability of cancer cells to avoid apoptosis and continue to proliferate is one of the fundamental hallmarks of cancer and is a major target of cancer therapy development (110). Apoptosis is the most common mechanism by which the body eliminates damaged or unneeded cells without local inflammation from leakage of cell contents. As the σRs have known apoptotic effects on tumors (86), a more detailed review on their anticancer effects follows.

##### σ_1_R

σ_1_R ligands cause a cell cycle arrest underlined by p27 accumulation. Studies indicate σ_1_Rs modulate cell regulating volume processes in physiological conditions, indicating that σ_1_Rs protect cancer cells from apoptosis. It appears that the σ_1_Rs modulate differentiation (1053). However, other findings suggest that the σ_2_Rs play a very significant role in σR associated toxicity (1072).

4-(N-benzylpiperidin-4-yl)-4-iodobenzamide [4-IBP], a selective σ_1_R agonist, has been used to investigate whether this compound modifies the migration and proliferation of human cancer cells. 4-IBP has weak antiproliferative effects on human U373-MG glioblastoma and C32 melanoma cells but induces marked concentration-dependent decreases in the growth of human A549 NSCLC and PC3 prostate cancer cells by eliciting apoptosis. The compound was also significantly antimigratory in all four cancer cell lines (1073). These results indicate that up regulation of the σ_1_R decrease growth and migration of malignant human cells *in vitro,* a finding that has been supported by investigations using cells from other species and non-malignant cell types (1074). These authors investigated the expression of σ_1_R in various human cancer cell lines in comparison to non-cancerous cell lines, using real time RT-PCR and by western blotting with a σ_1_R specific antibody. Also investigated were the effect of σ_1_R and σ_2_R drugs and a σ_1_R silencing construct. The results suggest σ_1_R plays a role in proliferation and adhesion of breast cancer cells (1074).

##### σ_2_R

Over expression of σ_2_R induces apoptosis (740). σ_2_R proteins are over expressed in several tumor cell lines, but the bimolecular mechanism of this over expression still needs further clarification, although two-photon confocal has shown σ_2_Rs are present in mitochondria, lysozomes, endoplasmic reticulum and plasma membranes (1075). There is a possibility that this over expression can be used with a radioligand to visualize in human bladder cancer specimens, then if a possible correlation could be established between σ_2_R over expression and tumor tissue stage and grade. In studies done so far, results demonstrate that σ_2_R protein is normally expressed in human bladder and over expressed in the case of high-grade transitional cell carcinomas (112), indicating this technique shows promise for staging of some cancers (111).

σ_2_R agonists induce apoptosis in drug-resistant cancer cells (1038), enhance the potency of DNA damaging agents, and down-regulates expression of p-glycoprotein mRNA (1076). σ_2_R agonists increase lysosomal membrane permeability in the early stages of σ_2_R-induced cell death (1077). Thus, σ_2_R agonists may be useful in treatment of drug-resistant cancers and the σ_2_R may serve as a novel signaling pathway to apoptosis (15,981). Further work has demonstrated that the σ_2_Rs are located in lipid rafts in the cell membrane, and these lipid rafts may play an important role in the mechanism of σ_2_R -induced apoptosis (982).

Several σ_2_Rs ligands have been shown to trigger apoptosis in pancreatic cancer cells. More importantly, σ_2_Rs ligands are internalized rapidly by the cancer cells and are capable of delivering other small-molecule therapeutics (1077).

A summary of references related to the expression of σRs are outlined in Table 7. A summary of the references that describe the molecular action of σRs are outlined in Table 8. A summary of the references describing σR binding are outlined in Table 9. A summary of the references describing the role of σRs in pathophysiology are outlined in Table 10.

**Table 7.** Reference Summary: Expression of sigma R (σR -regions/tissues).

Location of σR in tissues	Function(s)	Reference(s)
CNS: corpus striatum, nucleus accumbens		(61)
Brain: substantia nigra, pars compacta		(656)
CNS: dentate gyrus of hippocampus, facial nucleus, thalamic, hypothalamic nucei, straitum, cerebellum dorsal raphe nucleus and locus coeruleus		(171,765,766,302)
Hippocampal pyramidal cell layer, hypothalamus, central grey and red nucleus, pontine, cranial nerve nuclei, pontine nuclei, Pons – medulla, spinal cord – ventral and dorsal route ganglia		(761)
Brain: cortex limbic area amygdala		(762)
Brain: cerebellum		(763)
Brain: Medulla – pons, midbrain, cerebellum, thalamus, straitum, cortex, hippocampus		(78,79)
Brain: cerebral cortex, straitum, hippocampus, cerebellum		(767)
Brain: substantia nigra, central grey matter, oculomotor nuclei, cerebellum, nucleus accumbens, amygdale, olfactory bulb, hippocampus, motor cortex		(766,768)
CNS	Glu regulation, regulates excitotoxic effect of Glu	(386,417,425,447,450,451, 769,770,775,)
CNS	Regulation of Mn_2+_, Hg_2+_ and Pb_2+_ neurotoxicity.	(379)
Neurons: ependymocytes, oligodendrocytes and peripheral nervous system Schwann cells		(171,672,771–773)
CNS	σ_1_R agonist are antiamnesic, improve cognitive abilities	(27,181,212,365,376,602,753, 776–781,783,785)
CNS	Neuroprotection – two subtypes of σ_1_R may affect differentially the Glu-mediated NMDA neurotransmission in the terminal and origin regions of the mesolimbic and nigrostriatal DA-ergic systems. Also, functional interaction between σ_2_R and NMDARs in the hippocampus. σ_1_R agonist may protect neurons by mechanism involving anti-apototic protein bcl-2.	(360,786–788, )
CNS	σ_1_R initiates neurite outgrowth and sprouting. σ_1_R agonist potentiates neurite-sprouting by nerve growth factor. σ_1_R agonist may potentiate effects of BDNF and EGF	(32,41,164,176,177,185,803)
CNS	Mechanism of action of σR ligands in depression – regulation of Ca^2+^ or K^+^ signaling. Interacts with VDCC’s. Modulate Glu and 5-HT transmissions. May be a target for serotonin reuptake inhibitors.	(88,133,139,153,161,164–166,181,249–251, 376,377,664,828, 829)
Brain: frontoparietal cortex, cingulated cortex, dorsal striatum, nucleus accumbens	σ_1_R ligands affect iGluR subunit levels of mRNA and protein, differentially regulating levels of NMDA_2A_ and GluR_2_ in a regionally specific manner.	(15,380,802)
CNS	Activation of σ_1_Rs antagonize opioid analgesia whereas antagonists potentiate opioid analgesia. Excitatory amino acids have actions on σRs indicating action via the Glu system. Activation of spinal σ_1_R enhances NMDA-induced pain via PKC- and PKA-dependent phosphorylation of the NMDA receptor NR_1_ subunit	(458,483,681,881–883,894)
CNS	Agonist of σ_1_R and σ_2_R inhibit NMDA-stimulated DA release from motor and limbic areas of rat brain.	(896)
ANS: Parasympathetic intracardiac neurons	Cardiac excitation and rhythmic control	(756,920–922)
Ion Channel: hERG channel	Cardiac excitation and rhythmic control	(919–922)
Heart and vessels	Effects of σ_1_R mediated via PKC- and PKA dependent phosphorylation of the NMDA receptor, altered Na^+^ channels	(150,458,928,929)
Heart	σR ligands modulate contractility, Ca^2+^ influx and cardiac rate. σR activation prevent reperfusion contracture, increases pressure in left ventricle and improves survival of cardiac myocytes after ischemia and reperfusion. Activation of σR reversibly blocks the delay in outwardly rectifying K^+^ channels, conductance Ca^2+^ sensitive K^+^ channels and the M-current.	(923,930–932)
Peripheral arteries	σR agonist increase intracellular Ca^2+^ levels by stimulating IP3 production, modulating contractility.	(167)
Muscle	Dystonia	(764,938)
Osteoblasts	Act in conjunction with GluRs to affect cellular changes	(943,944)
Osteocytes, osteoclasts	Act in conjunction with GluRs to affect cellular changes	(492,497,765,945–947)
Lung: pulmonary nerves		(951)
Larynx, esophagus, mast cells		(516,957,958)
Airway passage	Excitation of GluRs may be important in airway inflammation and hyper reactivity observed in bronchial asthma	(440,959,960)
Airways	Antitussive	(194,733,961)
Pituitary, adrenal, testis and ovary		(676)
Endocrine system	Regulatory functions	(127,222,962)
Pituitary	Control of pituitary functions	(868)
Endocrine system	Antidiuretic hormone release	(968)
Posterior pituitary	Inhibit K^+^ channel function	(138,139)
Placenta		(976)
Spermatozoa	May affect signaling pathways in conjunction with PROG or prostaglandin E_1._	(976,977)
Ovary – follicles		(676)
Testis: ductuli efferentes, ductus epididymis, seminiferous tubules		(676)
Xenopus oocytes, neurohypophysis	Modulation of ion channels. Forms a immunoprecipitating complex with ion channels	(88)
Liver, localized to lipid rafts in rat liver phospholipid membranes, mitochondria		(92,113,980–983)
Kidney		(92)
Eye: retinal ganglion cells, inner nuclear membrane, inner segments of the photoreceptors, retinal pigment epithelial cells, retinal Mueller cells,	Neuroprotection against ganglion Glu toxicity, apoptosis, σ_1_R and σ_2_R binding activity stimulated during oxidative stress, important during lens development	(74,783,913,987,989–991,994,995)
Myenteric plexus of the guinea pig ileum	Regulation of ileal contractions, may be involved in regulating acid secretion in stomach	(314,747,998,999)
Gastrointestinal longitudinal muscle and myenteric plexus	Inhibit electrically or 5-HT-evoked contractions, stimulation of salivary secretion	(314,1001)
Vagus	Induce emesis	(1002–1005)
Human peripheral blood mononuclear cells, lymphocytes	σ2Rs inhibit lymphocyte activation. Potent immunoregulatory properties including induction of IL-10, suppression of IFN-γ and suppression of granulocyte colony stimulating factor.	(98,677,1010–1014)
Splenocytes	Lymphocyte proliferation, mitogen-induced IgG and IgM production, LPS-induced IL-I production	(98,1020)
Viral RNA	Regulate early steps in viral RNA replication	(1017,1018)
Host antitumor immunity	σR-dependent cytokine modulation. Ligand can induce apoptosis by changes in cytosolic Ca^2+^, ceramide and sphingolipid concentrations.	(1019,1013)
Neoplasia	Receptors overexpressed in many cancer tissues	(1022,1023)

**Table 8.** Reference Summary: Molecular Action of σRs.

Molecular action of σR	Tissue	Reference(s)
Glu regulation: regulates excitotoxic effect of Glu. two subtypes of σ1R may affect differentially the Glu-mediated NMDA neurotransmission in the terminal and origin regions of the mesolimbic and nigrostriatal DA-ergic systems. Functional interaction between σ2R and NMDARs in the hippocampus. σ1R agonist may protect neurons by mechanism involving anti-apototic protein bcl-2	CNS	(27,360,386,417,425,447,450,451, 769,770,775,786–788)
Regulation of Mn_2+_, Hg_2+_ and Pb_2+_ neurotoxicity	CNS	(379)
σ_1_R initiates neurite outgrowth and sprouting. σ_1_R agonist potentiates nerite-sprouting by nerve growth factor. σ_1_R agonist may potentiate effects of BDNF and EGF	CNS	(32,41,164,176,177,185,803)
Regulation of Ca^2+^ or K^+^ signaling. Interacts with VDCC’s. Modulate Glu and 5-HT transmissions.	CNS	(88,133,138,139,153,154,161,164--166, 181,249–251,377,821,829)
σ_1_R ligands affect iGluR subunit levels of mRNA and protein, differentially regulating levels of NMDA_2A_ and GluR_2_ in a regionally specific manner.	Frontoparietal cortex, cingulated cortex, dorsal striatum, nucleus accumbens	(15,380,802)
Activation of spinal σ_1_R enhances NMDA-induced pain via PKC- and PKA-dependent phosphorylation of the NMDA receptor NR_1_ subunit	CNS	(458,483,681,881–883,894)
Agonist of σ_1_R and σ_2_R inhibit NMDA-stimulated DA release from motor and limbic areas of rat brain.	CNS	(896)
Cardiac excitation and rhythmic control	Parasympathetic intracardiac neurons, hERG channel	(756,919–922)
Effects of σ_1_R mediated via PKC- and PKA dependent phosphorylation of the NMDA receptor, altered Na^+^ channels	Heart and vessels	(150,458,928,929)
σR ligands modulate contractility, Ca^2+^ influx and cardiac rate. σR activation prevent reperfusion contracture, increases pressure in left ventricle and improves survival of cardiac myocytes after ischemia and reperfusion. Activation of σR reversibly blocks the delay in outwardly rectifying K^+^ channels, conductance Ca^2+^ sensitive K^+^ channels and the M-current.	Heart	(923,930–932)
σR agonist increase intracellular Ca^2+^ levels by stimulating IP3 production, modulating contractility	Peripheral arteries	(167)
Ca^2+^ influx	Muscle	(764,938)
Act in conjunction with GluRs to affect cellular changes	Osteoblasts, osteocytes, osteoclasts	(492,497,943–948)
Modulate ion channels	Lungs	(194,440,733,959–961)
Regulatory functions. Control of pituitary functions. Antidiuretic hormone release. Inhibit K^+^ channel function.	Endocrine system, pituitary	(127,222,868,962,968)
May affect their signaling pathways in conjunction with PROG or prostaglandin E_1._	Spermatozoa	(976,977)
Modulation of ion channels. Forms a immunoprecipitating complex with ion channels	Xenopus oocytes, neurohypophysis	(88)
Neuroprotection against ganglion Glu toxicity, apoptosis, σ_1_R and σ_2_R binding activity stimulated during oxidative stress, important during lens development	Eye – retinal ganglion cells, inner nuclear membrane, inner segments of the photoreceptors, retinal pigment epithelial cells, retinal Mueller cells	(74,783,913,987,989–991,994,995)
Regulation of ileal contractions, may be involved in regulating acid secretion in stomach	Myenteric plexus of the guinea pig ileum	(314,747,998,999)
Inhibit electrically or 5-HT-evoked contractions, stimulation of salivary secretion	Gastrointestinal longitudinal muscle and myenteric plexus	(314,1001)
5-HT transmissions.	Vagus	(1002–1005)
σ2Rs inhibit lymphocyte activation. Potent immunoregulatory properties including induction of IL-10, suppression if IFN-γ and suppression of granulocyte colony stimulating factor.	Human peripheral blood mononuclear cells, lymphocytes	(98,677,1010–1014)
Lymphocyte proliferation, miogen-induced IgG and IgM production, LPS-induced IL-I production	Splenocytes	(98,1020)

**Table 9.** Reference Summary: σR Binding (679, 680).

Drug	Target tissue
(+)-pentazocine [PTZ]	Sigma_1_ Sigma_2_ ligands
Haloperidol [Haldol®]	Sigma_1_ Sigma_2_ ligands
1,3 di-o-tolyl-guanidine [DTG]	Sigma_1_ Sigma_2_ ligands
(+)-3-PPP [preclamol]	Sigma_1_ Sigma_2_ ligands
(+)-SKF 10,047	Sigma_1_ Sigma_2_ ligands
(+)-pentazocine [PTZ]	Sigma_1_ Sigma_2_ ligands
Phencyclidine	Sigma_1_ Sigma_2_ ligands
Dextromethorphan [DEX]	Sigma_1_ ligands
(+)-cyclazocine	Sigma_1_ ligands
BD1047	Sigma_2_ ligands
BD1063	Sigma_2_ ligands

**Table 10.** Reference Summary: Role of σRs in Pathophysiology.

Tissue type – disorder		Function or role in pathology	Reference(s)
CNS	Memory loss	σR ligands may be antiamnesic, improve cognitive abilities	(181,212,365,376,602,753,776–783,785)
CNS	Neurodegeneration	Delays cerebral artery occlusion-induced neurodegeneration and white matter injury. σ_1_R agonist protect neurons by a mechanism involving the anti-apoptotic protein bcl-2. Initiation of neurite outgrowth and sprouting. Overstimulation of σR is mediated via Glu, particularly NMDAR leading to osmotic damage, apoptosis and necrosis.	(27,32,41,164,176,177,185,382,384–386,394,412,438,511,787,788)
CNS	Schizophrenia	σ_1_R polymorphism is associated with increased rick of schizophrenia and differential activation of PFC and the severity of AD.	(44,344)
CNS	Depression, stress	σR, Glu, 5-HT neurotransmission and Ca^2+^ regulation. Reduced brain σ_1_R exacerbates heart failure. Regulation of Ca^2+^ or K^+^ in neurotransmission.	(26,57,88,133,138,139,153,154,161,164,165,181,249–251,326,376,805–810,828,829)
CNS	Psychosis	Selective σ_1_R ligans potentially stimulate adrenocorticotropic hormone release, regulation of neuroendocrine system in brain.	(868–870)
CNS	Seizures	Complex involvement of 5-HT_2_, DA and σRs. Increase in neuropeptide biosynthesis may play a compensatory anticonvulsive role. Seizure activity by overstimulation of σR is mediated via Glu, particularly NMDAR.	(407,538,872,873,906–908)
CNS	Pain	Activation of σ_1_Rs antagonize opioid analgesia whereas antagonists potentiate opioid analgesia. Excitatory amino acids have actions on σRs indicating action via the Glu system. Activation of spinal σ_1_R enhances NMDA-induced pain via PKC- and PKA-dependent phosphorylation of the NMDA receptor NR_1_ subunit.	(483,458,681,881–883,894)
CNS	Addiction	Both cocaine and METH exhibit a significant affinities for σRs. Agonist of σ_1_R and σ_2_R inhibit NMDA-stimulated DA release from motor and limbic areas of rat brain.	(872,896–898)
Heart and blood vessels	Heart failure	Reduced σ_1_Rs density in depression decreases heart rate via the sympathetic stimulation in the autonomic nervous system. Reduction of brain σ_1_Rs also contribute to sympathetic hyperactivation of the heart via altered Na^+^ channels. Activation of σ_1_R depresses the excitability of intracardiac neurons causing changes in beating frequencies, which are followed by irregular contractions. σRs are involved in the regulation of coronary and peripheral arterial vascular tension.	(150,751,809,810,923,927–933)
Muscle	drug-induced dystonia	High affinity of some neuroleptics for these sites suggests their possible involvement in some σ_2_R-mediated side effects.	(764,938)
Bone	Bone	All osteoblasts, osteocytes and osteoclasts express one or more of the GluR subunits, including NMDARs. Possible that σRs are involved in normal bone function as well as in disease states.	(946,943,944,947,492,945,497,948)
Lung	Asthma	The presence σRs in the airway structures such as the larynx, esophagus and mast cells also implicate the GluRs (and probably the σRs) in the mediation of asthmatic episodes.	(440,516,957–960)
Endocrine	Drug induced syndrome of inappropriate antidiuretic hormone release.	Interaction with some neuroleptic agents and the posterior pituitary σR ligands can inhibit K^+^-channel function.	(138,139,968)
Endocrine	Diabetes mellitus	Dysfunctional islet cells	(435,486–488,499,523,524,669,969,970,971,512,972,973)
Eye	Glaucoma, diabetes retinopathy, retinal ischemia due to central artery occlusion, anterior ischemic optic neuropathy, optic neuritis, optic nerve trauma	Possible neuroprotection by σR ligands against ganglion cell Glu toxicity. Late-onset inner retinal dysfunction in mice lacking σ_1_R. Exposure of lens cells to σR antagonists has been shown to lead to growth inhibition and pigment granule production implying importance during lens development. Gene silencing of the σ_1_R induces cell death.	(987,988,992–995)
Gastrointestinal	Emesis	σRs induce emesis in a number of species mediated centrally via the vagus.	(1002–1004)
Immune system	Graft versus host reactions and delayed-type hypersensitivity granuloma formation. Immune dysregulation	σ_2_Rs inhibit T lymphocyte activation. σR ligands have potent immunoregulatory properties, including the induction of IL-10 and the suppression of IFN-γ and granulocyte colony stimulating factor [GM-CSF].	(98,1012–1014)

## Conclusions

The neuropharmacological properties of σ_1_R ligands relate to the neuron modulatory role of σ_1_R. σ_1_Rs act as intracellular amplifiers for signal transductions involving IP3R and modulate neurotransmitter systems (mainly through NMDA receptors). σ_1_Rs and ion channels may play an important role in neuroplasticity processes. σ_1_R ligands are highly active when a pharmacological or pathological imbalanced state arises.

The combined administration of σ_1_R receptor ligands and medications with a known therapeutic effect has been shown to improve these effects due to the modulatory role of σ_1_R receptors resulting in the need for lower doses to reach therapeutic concentrations. Of particular interest is the non-linear dose response curve of σ_1_R agonists in *in vitro* experiments, in which σ_1_R agonists are active, e.g. learning and memory processes, depression (1078) and anxiety. These findings imply that researchers should take hormesis into account in order to design informative experiments or clinical trials with σ_1_R agonists. For example, the σ_1_R SA4503 agonist attenuates or enhances the effects of methamphetamine depending on the dose (682).

The most promising therapeutic targets for σ_1_R antagonism are nociception and some deleterious effects of certain drugs of abuse such as cocaine, methamphetamine and ethanol (1079). Many drugs used routinely show affinity for σ_1_R receptors and exert the same effects as other more selective σ_1_R ligands in many behavioral tests and *in vitro* assays. Therefore, the therapeutic properties of these drugs might be due, at least in part, to their interaction with σ_1_R receptors.

The involvement of σRs in the cellular pathophysiology of cancer is apparent from the high density of σ_1_R and σ_2_R-binding sites found in various tumor cell lines and tissues. Consequently, σR drugs have been suggested to be potentially useful tumor imaging agents.

The ability of σ_2_R drugs to inhibit tumor cell proliferation through mechanisms that may involve apoptosis, intracellular Ca^2+^ and sphingolipids have been investigated, and such findings may lead to the development of σ drugs as cancer therapeutic agents. It is possible that an increase in σ_2_R expression is a significant event in transition from normal to malignant cells. Further research would be interesting to determine whether σR are involved in other metastatic cell behaviors such as adhesion, secretion, motility and invasion.

The interaction of σRs and other neurotransmitters is complex. As has been discussed in this review, σRs are intimately involved with the glutamate system, and are probably an essential part of the expression of excitotoxicity (1080). Other interactions with opiates, neurosteroids, serotonin, dopamine and cannabinoids have been difficult to fully elucidate due to the biphasic nature of dose response curves and the large combination of potential effects. In addition, as most work is done in *in vitro*, doses are often excessive and may reflect an overexposure that would not be seen in the *in vivo* situation. Even though the interaction of σRs with various tissues is complex, it is apparent that σRs play a central role in neurotransmission and apoptosis. The development of new knockout mice and transgenic initiatives will be important to further research.
